# Glioma oncogenesis in the Constitutional mismatch repair deficiency (CMMRD) syndrome

**DOI:** 10.1093/noajnl/vdae120

**Published:** 2024-07-11

**Authors:** Lea Guerrini-Rousseau, Jane Merlevede, Philippe Denizeau, Felipe Andreiuolo, Pascale Varlet, Stéphanie Puget, Kevin Beccaria, Thomas Blauwblomme, Odile Cabaret, Nadim Hamzaoui, Franck Bourdeaut, Cécile Faure-Conter, Martine Muleris, Chrystelle Colas, Tiphaine Adam de Beaumais, David Castel, Etienne Rouleau, Laurence Brugières, Jacques Grill, Marie-Anne Debily

**Affiliations:** Department of Children and Adolescents Oncology, Gustave Roussy, Villejuif, France; Molecular Predictors and New Targets in Oncology, INSERM U981, Team “Genomics and Oncogenesis of pediatric Brain Tumors,” Gustave Roussy, Université Paris-Saclay, Villejuif, France; Molecular Predictors and New Targets in Oncology, INSERM U981, Team “Genomics and Oncogenesis of pediatric Brain Tumors,” Gustave Roussy, Université Paris-Saclay, Villejuif, France; Clinical Genetics, Rennes University Hospital, Rennes, France; Neuropathology and INSERM UMR1266 IMA-Brain, GHU-Paris Psychiatry and Neuroscience, Sainte-Anne Hospital, Paris, France; Neuropathology and INSERM UMR1266 IMA-Brain, GHU-Paris Psychiatry and Neuroscience, Sainte-Anne Hospital, Paris, France; Neurosurgery, Necker Hospital, Paris University, Paris, France; Neurosurgery, Necker Hospital, Paris University, Paris, France; Neurosurgery, Necker Hospital, Paris University, Paris, France; Department of Medical Genetics, Gustave Roussy, Villejuif, France; Service de Génétique et Biologie Moléculaires, Hôpital Cochin, APHP Centre Université de Paris, Paris, France; Inserm UMR_S1016, Institut Cochin, Université de Paris, Paris, France; Translational Research in Pediatric Oncology (RTOP), INSERM U830 Laboratory of Genetics and Biology of Cancers, SIREDO: Care, Innovation, and Research for Children, Adolescents and Young Adults with Cancer, Curie Institute, Paris University, Paris, France; Pediatric Hematology and Oncology Institute (IHOPE), Centre Leon Berard, Lyon, France; Centre de Recherche Saint-Antoine, Sorbonne Université, Paris, France; Département de Génétique, Institut Curie, Université Paris Sciences Lettres, Paris, France; Department of Children and Adolescents Oncology, Gustave Roussy, Villejuif, France; Molecular Predictors and New Targets in Oncology, INSERM U981, Team “Genomics and Oncogenesis of pediatric Brain Tumors,” Gustave Roussy, Université Paris-Saclay, Villejuif, France; Department of Medical Genetics, Gustave Roussy, Villejuif, France; Department of Children and Adolescents Oncology, Gustave Roussy, Villejuif, France; Molecular Predictors and New Targets in Oncology, INSERM U981, Team “Genomics and Oncogenesis of pediatric Brain Tumors,” Gustave Roussy, Université Paris-Saclay, Villejuif, France; Department of Children and Adolescents Oncology, Gustave Roussy, Villejuif, France; Molecular Predictors and New Targets in Oncology, INSERM U981, Team “Genomics and Oncogenesis of pediatric Brain Tumors,” Gustave Roussy, Université Paris-Saclay, Villejuif, France; Molecular Predictors and New Targets in Oncology, INSERM U981, Team “Genomics and Oncogenesis of pediatric Brain Tumors,” Gustave Roussy, Université Paris-Saclay, Villejuif, France; Département de Biologie, Université Evry, Université Paris-Saclay, Evry, France

**Keywords:** cancer predisposition, CMMRD, constitutional mismatch repair deficiency, glioblastoma, mutation burden, mutational signatures

## Abstract

**Background:**

Constitutional mismatch repair deficiency (CMMRD) is a cancer predisposition due to biallelic mutations in one of the mismatch repair (MMR) genes associated with early onset of cancers, especially high-grade gliomas. Our aim was to decipher the molecular specificities of these gliomas.

**Methods:**

Clinical, histopathological, and whole exome sequencing data were analyzed in 12 children with genetically proven CMMRD and a high-grade glioma.

**Results:**

PDL1 expression was present in immunohistochemistry in 50% of the samples. In 9 patients, the glioma harbored an ultra-hypermutated phenotype (104–635 coding single nucleotide variants (SNV) per Mb, median 204). Driver mutations in *POLE* and *POLD1* exonuclease domains were described for 8 and 1 patients respectively and were always present in the mutation burst with the highest variant allele frequency (VAF). The mutational signatures were dominated by MMR-related ones and similar in the different mutation bursts of a same patient without subsequent enrichment of the mutation signatures with POL-driven ones. Median number of coding SNV with VAF above one of the driving polymerase mutation per Mb was 57 (17–191). Our findings suggest that somatic polymerase alterations does not entirely explain the ultra-hypermutant phenotype. *SETD2*, *TP53*, *NF1*, *EPHB2*, *PRKDC,* and *DICER1* genes were frequently mutated with higher VAF than the deleterious somatic polymerase mutation.

**Conclusions:**

CMMRD-associated gliomas have a specific oncogenesis that does not involve usual pathways and mutations seen in sporadic pediatric or adult glioblastomas. Frequent alterations in other pathways such as MAPK may suggest the use of other targeted therapies along with PD1 inhibitors.

## Lay Summary

Patients with mutations in genes responsible for repairing damaged DNA are at a higher risk of developing cancer at an early age. Gliomas are a type of aggressive brain cancer that can occur in these patients. The authors of this study wanted to understand the differences between gliomas in patients with and without this specific genetic condition. To do this, they reviewed 12 children with the genetic condition who developed gliomas. Their results showed that 9 patients had tumors with a higher than expected number of mutations (ultra-mutated). The genes that are commonly mutated in these patients were not identical to those that are commonly known to occur without this genetic condition.

**Key Points**

- CMMRD-associated gliomas have a specific oncogenesis in addition to the ultra-hypermutated phenotype.
- POLE/D1 mutations may not be the only mechanism driving the ultra-hypermutated phenotype.
- Mutations in the MAPK pathway occur frequently, with higher VAF than POL mutation.

**Importance of the Study**

CMMRD syndrome is one of the most frequent predisposition to high-grade gliomas in children and adolescents. Tumors are characterized by an ultra-hypermutated phenotype. Mutations do however no occur randomly and do not necessarily affect classical glioma-related genes. The description of this specific oncogenesis could be helpful to define new therapeutic targets to improve the current standard of therapy with radiation and immunotherapy which is not efficient in every cases. Unlike other large genes, NF1 and other MAPK pathway genes are frequently affected by mutations at VAF higher than the polymerase one suggesting MEK inhibitors as potential candidates for future combination studies.

The contribution of defective mismatch repair (MMR) to cancer development has been acknowledged now for more than 2 decades.^1^ Heterozygous (monoallelic) germline mutations in the MMR genes (*MLH1*, *MSH2*, *MSH6,* and *PMS2)* cause the autosomal-dominant Lynch syndrome (LS), which mostly predisposes to colorectal and endometrial cancers after the fourth decade of life.^2,3^ Biallelic (homozygous or compound heterozygous) germline mutations in one of the 4 MMR genes is a rare autosomal recessive inherited cancer predisposition syndrome called Constitutional Mismatch Repair Deficiency (CMMRD), first described in 1999.^4–6^ Due to the constitutional defect in MMR capacity, individuals with biallelic germline mutation in a MMR gene have a high cancer risk already in childhood and adolescence including (i) hematological malignancies, (ii) brain/central nervous system tumors, and (iii) colorectal and other cancers that are typically seen in LS-patients at a later age.

The criteria for CMMRD genetic testing in patients with cancer have been published by Wimmer et al. in 2014 ^7^. Many of the CMMRD patients, show features reminiscent of neurofibromatosis type 1 (NF1), particularly multiple café-au-lait maculae (CALM).^8^ As malignant brain tumors are rare in NF1, in face of a malignant tumor in a child with CALM, CMMRD should be ruled out before considering the diagnosis of NF1, especially now that different therapeutic strategies are applied.^9,10^ In addition to the presence of a family history of cancer and/or consanguinity, specific radiological or histological features can also guide the clinician towards the presence of CMMRD. Contrary to classical glioblastomas, CMMRD-associated gliomas are characterized by a loss of expression of at least one MMR protein detected by immunostaining in both tumor and normal cells, and may be associated with histological features of a malignant glial heterogenous population with giant multinucleated and pleomorphic cells in many of them.^11^ Radiological findings of metachronous multiple brain lesions and/or brain vascular malformations have been described in CMMRD-population with a high rate of incidence.^11,12^ These features are not adjacent to the brain tumor and not related to the tumor treatment and are usually asymptomatic.^7,12^ Finally, CMMRD-associated tumors are characterized by a high mutation rate^13,14^ leading to microsatellite instability and to the expression of a large amount of neoantigens representing an opportunity for immunotherapy. Indeed, MMR-deficient tumors are more responsive to PD-1 inhibition than MMR-proficient tumors.^15–17^

One of these above-mentioned characteristics must question the possibility of a cancer predisposition syndrome with CMMRD and justify additional investigations on appropriate microsatellite instability analysis, functional assays,^18–21^ and MMR genotyping on the patient’s constitutional DNA. In these ultra-hypermuted tumor cases, a secondary somatic event such as an inactivating mutation in *POLE* or *POLD1* genes is considered to be responsible for the mutation burst(s) leading to the ultra-hypermutated phenotype. Apart from this event, little is known about specific oncogenic pathways involved. Defining the oncogenesis of the tumors occurring in the context of this predisposition syndrome is of paramount importance in order to find out specific mutational events that could be screened in cell-free circulating tumor DNA of affected children or become potential new therapeutic targets. In this study, we analyzed comprehensively a series of 12 high-grade gliomas (HGG) from CMMRD-patients in order to describe the specific phenotypic and biologic characteristics of these tumors in the context of this predisposition to compare them to the other sporadic pediatric HGGs.

## Patients and Methods

### Patients

CMMRD was suspected in patients who meet the CMMRD diagnostic criteria established by the European Care for CMMRD (C4CMMRD) consortium. They were referred to the oncogenetic clinics and a germline molecular analysis restricted to the MMR genes was performed after each patient/parent gave informed consent to perform the genetic testing. After confirmation of the underlying predisposition, these 12 patients were registered in the C4CMMRD (IRB00003888) registry database that collected their clinical data and family history. All these patients were described in the previous report of the C4CMMRD consortium on brain tumors.^11^ For an easy identification, we have kept for each patient, the same numbering as in this publication. In addition to the basic clinical data usually studied, the data analyzed for each patient consisted of a central pathology review, functional tests in peripheral blood lymphocytes, and analysis of whole exome sequencing (WES) from blood and tumor results.

### Pathology Review

A histological central review by 2 independent expert pediatric neuropathologists (PV, FA) was performed according to the 2016 WHO guidelines^22^ using the available paraffin-embedded tumor sections, including hematoxylin–eosin–saffron (HES) staining for morphological description, specific immunohistochemical stainings, and pathological assessment of immune infiltrates.

Immunolabeling was conducted using an OMNIS automate (DAKO, Agilent). A standard pretreatment protocol pH9 was followed by a room temperature primary antibody incubation. The following primary antibodies were used: MLH1 (prediluted, clone E505, Agilent), MSH2 (prediluted, clone FE11, Agilent) or MSH6 (prediluted, clone EP49, Agilent) or PMS2 (prediluted, clone EP51, Agilent). For PD1, the immunolabeling was realized with a Ventana Discovery XT. A standard pretreatment protocol pH9, was followed by a 32 min incubation (37°C) of the primary PD1 antibody (dilution 1/50, clone NAT-105, Diagomics, Blagnac, France). Lymphocyte infiltrates were evidenced with anti-CD8 staining (prediluted, clone C8/144B, Dako); immunohistochemical staining analysis was considered positive in case of diffuse staining or tertiary lymphocyte structures (islands isolated in the tumor tissue).

PD1 expression was evaluated semi-quantitatively using a cutoff of <5% (negative) of tumoral PD1 positive cells versus >5% (positive). Staining for p53 was considered as positive if more than 10% of nucleus of tumors cells were positive.

### Whole Exome Sequencing (References are Provided as Supplementary File)

The genomic DNA of the tumor was extracted from frozen tissue (Qiagen, Crawley, UK). Matched constitutional DNA was extracted from blood samples (Qiagen, Crawley, UK). Genomic DNA of pairs tumor—blood samples was captured using Agilent in-solution enrichment methodology (SureSelect XT Clinical Research Exome, Agilent) and subjected to paired-end 75 bases massively parallel sequencing on Illumina HiSeq4000. Quality of reads was evaluated using FastQC (http://www.bioinformatics.bbsrc.ac.uk/projects/fastqc/).

Adaptor sequences and low quality bases were trimmed from raw reads ends with Trimommatic and reads were then aligned to the reference human genome hg19 using BWA-mem algorithm; reads with mapping quality <20 were excluded. Polymerase chain reaction duplicates were removed with Picard MarkDuplicates. Local realignment around indels was performed using GATK (Genome Analysis ToolKit) RealignerTargetCreator and IndelRealigner. Base quality score recalibration was performed using GATK BaseRecalibrator. Bases with a Phred-based quality score ≤20 were ignored. The mean sequencing depth in the targeted regions was 153× (SD 27×) in tumor and 92× (SD 26×) in the blood samples. More details about read processing are provided in Supplementary Material. Somatic and germline single nucleotide variants (SNV) and indels were called with VarScan2. Remaining variants were annotated with Annovar. Parameters used for variant detection, annotation as well as the version of the tools are detailed in Supplementary Data.

Mutational signatures were ascertained by grouping somatic substitutions on the basis of their 5ʹ and 3ʹ bases into 96 possible trinucleotide categories.^13^ Since several well-known signatures were expected (DNA MMR deficiency, *POLE* mutation, . . . ), a supervised approach, namely SigProfiler Assignment^23^ was chosen to identify the mutational signatures acting in each patient, as well as their contribution. The 67 signatures from COSMIC were used to reconstruct the mutational profile of each patient. Several metrics were used to compare the original spectrum with the reconstructed one and indicated very good correlations between both profiles. Finally, joint inference of tumor-intra heterogeneity and mutational processes was performed using CloneSig. Between 1 and 5 clones were identified by patient, a limited number given the high number of mutations. For example, the patient 4A has around 35,000 somatic coding mutations but only 3 clones. Except one patient, all patients had one major clone and one or several minor clones (Supplementary Figures 3 and 4). The signature activity of the different mutational processes acting in the tumors are very similar in the different clones (Supplementary Figure 4). This analysis indicates that POLE/POLD1 did not introduce clones with different mutational profiles and that, even though the mutation rate is very high, the mutation processes are not random but instead still well organized.

Copy Number Variations (CNV) and Loss Of Heterozygosity (LOH) analysis were assessed using Sequenza^24^ as well as an in house method later packaged in EaCoN (https://github.com/gustaveroussy/EaCoN). More details are provided in Supplementary Data.

DrGaP was used to identify potential driver genes.^25^

## Results

### Patients and General Characteristics

Twelve patients including 2 siblings, from 11 nuclear families, with complete clinical, radiological, and pathological annotations as well as constitutional and tumor WES data were identified. There were a majority of boys (*n* = 9). Overall, 22 malignancies were diagnosed in these 12 patients: 16 HGGs including 14 glioblastomas and 2 anaplastic astrocytomas grade III (9 as first, 5 as second, and 2 as third neoplasm), 5 lymphoblastic lymphomas (4 TLL and 1 BLL) and 1 lieberkuhn adenocarcinoma of the colon (Supplementary Table 1). The HGG was the first tumor observed in 9/12 children. The median age at onset of the first tumor and first HGG were 6.5 [3–13] and 7.5 [3–14] years, respectively. Six patients had multiple brain malignancies: 2 developed multifocal disease at the time of the initial diagnosis, and 4 patients had asynchronous glioblastomas. Multiple CALMs (>10) were observed in all patients and 4 patients had also hypochromic spots. Consanguinity was reported in 64% (7/11) of families. MMR gene alterations were identified only in *PMS2* (*n *= 7 patients) and *MSH6* (*n *= 5 patients) (Table 1 and Supplementary Table 2). The MMR germline mutations were homozygous in 10/12 children (9 families) and compound heterozygous in 2 patients (patients 26 and 31). All patients had pathogenic germline mutations in MMR genes, except one (patient 11) who presented a biallelic variant of unknown significance (VUS), but an ultra-hypermutated phenotype and abnormal functional test. In 4 unrelated patients (patients 1, 5, 14, and 19) all from Tunisian families, the same *PMS2* splice variation was observed, suggesting a common founder mutation. The diagnosis of CMMRD was also confirmed by functional tests results in 9 out of 10 (Supplementary Table 2) and/or loss of expression of the MMR protein detected by immunohistochemistry in non-neoplastic cells in 11 of 12 tested (Table 1).

**Table 1.** Histology and Immunohistochemistry of the Tumors. Immune Infiltrates Were Evaluated as Previously Published (Mackay et al., Cancer Cell 2018): Score 0 Means Absence of CD8 Positive Cells, Score 1 Means Moderate T Lymphocyte Infiltrates, With Isolated Cells in the Proliferation, With Occasional and Focal Lymphoid Islands in the Most Aggressive Parts of the Tumor. PDL1 Staining is Graded According to the Percentage of Positive Tumor Cells: 2 for >50%, 1 for <50% of Positive Tumor Cells, 0 for <5% of Positive Tumor Cells. NA, not Available. GBM, Glioblastoma

Pt	MMR gene mutated	Histology	MMR proteins	ATRX loss	P53 nuclear staining	PDL1 score 0–2	CD8 infiltrates
1	*PMS2*	GBM IDH-wt (with few giant multi-nucleated cells)	PMS2 loss	1	1	1	1
4B	*MSH6*	GBM IDH-wt (with few giant multi-nucleated cells)	MSH6 loss	1	1	2	1
4A	*MSH6*	GBM IDH-wt (with few giant multi-nucleated cells)	MSH6 loss	1	1	0	1
5	*PMS2*	GBM (with few giant multi-nucleated cells)	PMS2 loss	1	1	0	1
11	*MSH6*	GBM (with few giant multi-nucleated cells)	MSH6 loss	0	1	1	0
14	*PMS2*	GBM (without giant cells)	PMS2 loss	1	1	1	1
19	*PMS2*	AA, IDH-wt (with giganto-cellular features)	PMS2 loss	0	1	2	1
23	*MSH6*	GBM (with few giant multi-nucleated cells)	MSH2 loss w/o MSH6 loss	1	NA	0	0
24	*PMS2*	GBM (with few giant multi-nucleated cells)	PMS2 loss	1	1	0	1
26	*PMS2*	AA IDH-wt	PMS2 loss	1	0	0	0
30	*MSH6*	GBM	NA	NA	NA	NA	NA
31	*PMS2*	GBM (with few giant multi-nucleated cells)	PMS2 loss	1	1	1	0

### Histological and Immunohistochemical Characteristics

**Figure 1.**
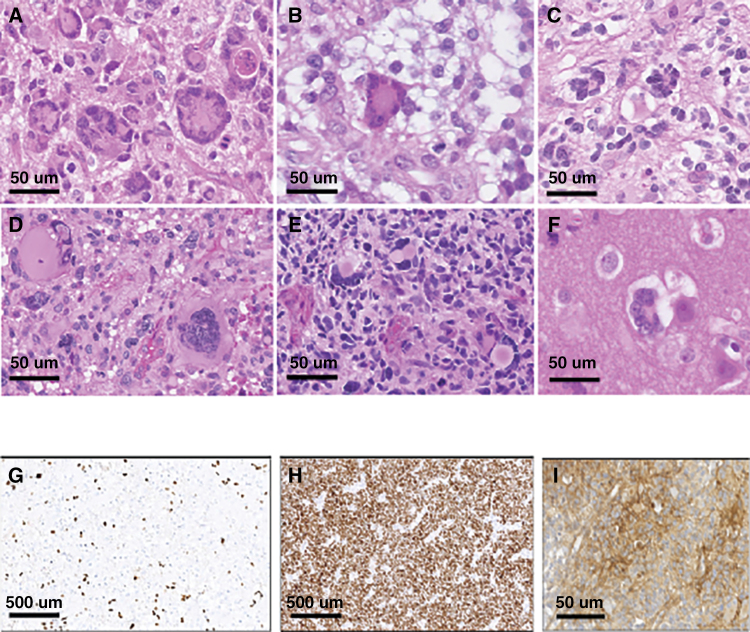
Representative histological and immunohistochemical characteristics of CMMRD. (A–F) Hematoxylin–Eosin–Safranin (HES) staining showing at high magnification the presence of giant and multinucleated tumor cells (observed in 8/12 tumors analyzed). (G) Loss of ATRX expression in the tumor cells (observed in 9/11 tumors analyzed). (H) Wide spread and strong nuclear positivity for *p53* protein (observed in 9/10 tumors analyzed). (I) Membranous staining of the tumor cells for PDL1 (observed in 6/11 tumors analyzed).

For each patient, one HGG was analyzed (Supplementary Table 1). The histopathological diagnosis was glioblastoma in 10, and anaplastic astrocytoma grade III, IDH-wildtype in the 2 other patients (including one with angiocentric features) (Table 1). The main histopathological characteristic was the frequent (*n* = 8) identification of giant tumor cells ranging from 2% to 30% of the tumor cells (Figure 1A–F). All tumors presented severe nuclear atypia.

Immunohistochemistry was performed in the 11 available formalin-fixed paraffin blocks of tumor samples and showed that 9/11 tumors exhibited ATRX loss (Figure 1G). Among these 9 samples, 8 were associated with an *ATRX* mutation. One additional tumor showed an *ATRX* mutation without loss of staining on IHC. Immunohistochemistry was positive for p53 in all but one patient tested (9/10 cases), all exhibiting at least one mutation (Figure 1H). All *TP53* mutations considered were deleterious, located in the DNA-binding domain and were described in COSMIC. Six of 11 tumors showed a positive staining for PDL1 (Figure 1I) and 7/11 tumors had a significant lymphocytic infiltrate. Loss of expression of PMS2 protein was observed in both tumor and non-neoplastic cells for all the 7 patients with a biallelic germline *PMS2* mutation. Among the 5 patients with a biallelic germline *MSH6* mutation, 4 patients had a loss of expression of the MSH6 protein in the tumor and in non-neoplastic cells. The last patient showed a loss of expression of the MSH2 protein in the tumor and in non-neoplastic cells despite having a homozygous MSH6 likely pathogenic variant and an ultra-hypermutated tumor possibly through the destabilization of the heterodimer with MSH6 (Table 1).

### Whole Exome Sequencing and Mutation Burden

**Figure 2.**
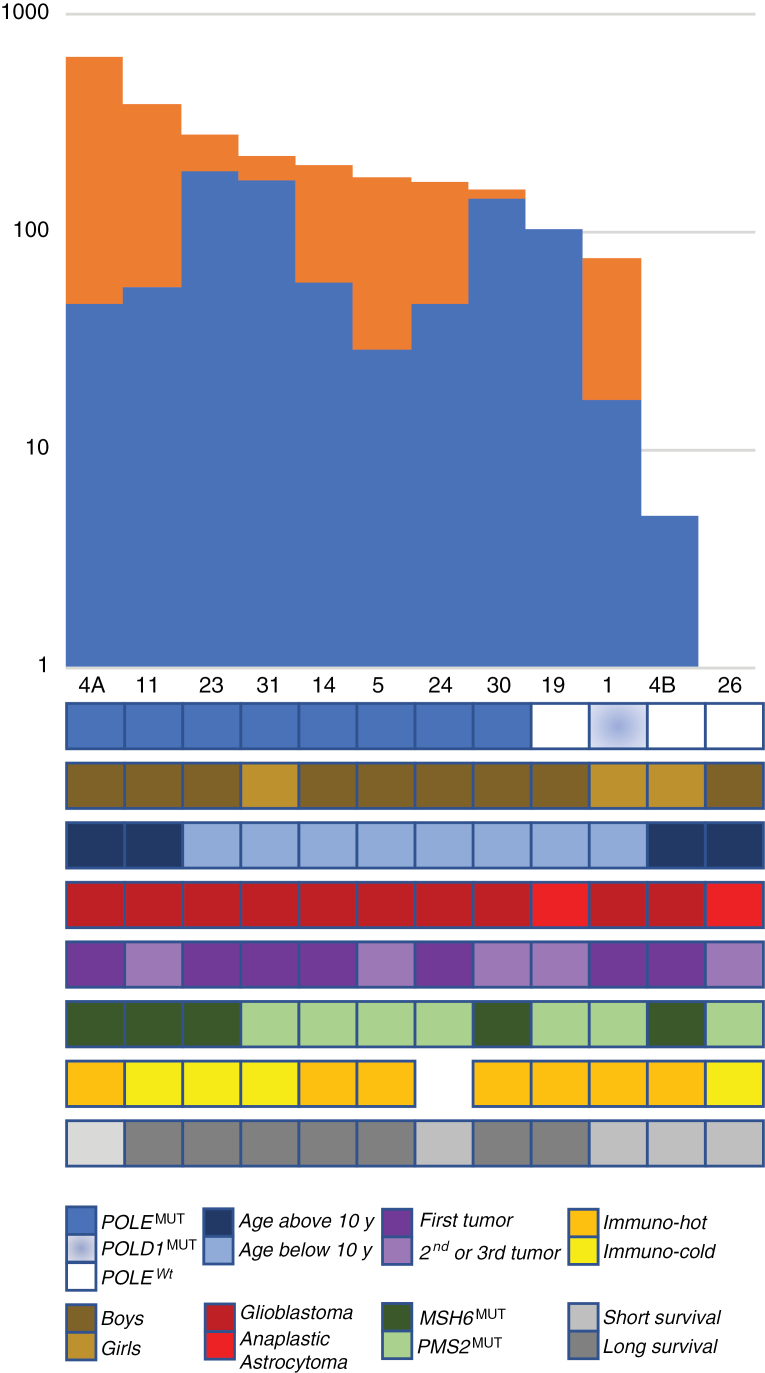
Tumor mutation burden and clinical characteristics. Upper Panel. Number of coding SNV/Mb are shown in a histogram in a log scale (y-axis). Coding SNV/Mb with higher VAF than the one of the driving *POLE/POLD1* mutation are represented in blue and coding SNV/Mb with lower VAF than the one of the driving *POLE/POLD1* mutation in orange. Lower Panel. Heatmap representing clinical parameters of CMMRD patients: age of the patient at diagnosis of the glioma, sex, histology, the ranking of the tumor as first or subsequent tumor, MMR mutated gene, immuno-hot and immune-cold tumors defined according to the importance of the CD8 positive lymphocyte infiltrates, current status of the patient at the time of the study.

A median of 9425 coding SNV [71–34283], corresponding to a tumor mutation burden (TMB) of 174 [range 1–635] coding SNV/Mb (Supplementary Table 3) was identified by WES. Three of the 12 patients (patients 1, 4B, and 26) had less than 100 somatic coding SNV/Mb, which is considered as the threshold for the ultra-hypermutant phenotype. Children with the highest number of mutations were not older or not less likely to be longer survivors (Figure 2). The number of mutations was higher in the tumors of patients with *MSH6* biallelic mutations compared to those with *PMS2* biallelic mutation as the 3 patients with the highest TMB had *MSH6* biallelic mutations but this difference did not reach statistical significance (median of 279 vs 170 coding SNV/Mb, respectively, *P* = NS, Mann Whitney Wilcoxon test). HGG occurring as second or subsequent neoplasm did not show higher TMB than those occurring as first neoplasm (Figure 2). Tumors considered as “immuno-hot,” that is with significant lymphocytic infiltration, were not correlated with higher TMB either.

At least one *POLE* somatic mutation was described in 9/12 patients. We selected 8 functionally validated inactivating *POLE* mutation, that will be considered as the potential co-driver mutation (Supplementary Table 4). One of the 4 patients without *POLE* co-driver somatic mutation had a *POLD1* mutation (patient 1) and the patient with a TMB just above the threshold of mutation defining ultra-hypermutator phenotype did not harbor neither *POLE* nor *POLD1* alterations (patient 19).

In the analysis of hypermutant cancers, mutations burst are usually considered according to their variant allele frequency (VAF), assuming that the mutations with the highest VAF occurred before the ones with lower VAF.^13,14^ This allowed these authors to describe different profiles of mutation burst associated with different mechanisms of mutation accumulation. As *POLE* mutation is supposed to trigger the ultra-hypermutation phenomenon in CMMRD patients,^13,14^ we computed the number of mutations with a VAF above (blue) or below (orange) the one of the driving *POLE/POLD1* mutation identified, assuming that the former were occurring earlier than the *POLE/POLD1* mutation (Figure 2). We could make this assumption only at 2 conditions: (1) the absence of significant clonal selection, (2) the absence of significant copy number changes. CNVs in this study were limited in CMMRD genomes as previously reported^13^ (Supplementary Figure 1). Based on the results obtained by Sequenza and EaCoN, there were indeed no CNV involving *POLE* or *POLD1* for any patient, in particular for the mutated polymerase. Four patients (patients 19, 23, 30, 31) had a number of mutations with higher VAF than the driving *POLE* mutation superior to the classical threshold for ultra-hypermutation (Figure 2). The number of mutations occurring supposedly after the driving *POLE* or *POLD1* in 9 patients increased from 0.09 to 12.33-fold, with a median of 2.6-fold. This could suggest that the ultra-hypermutation phenomenon may start before (or even independently in some cases) the occurrence of the *POLE* mutation or that there is a strong mechanism for selecting these specific mutations during oncogenesis.

### Clonality Analysis of HGG in CMMRD Patients

**Figure 3.**
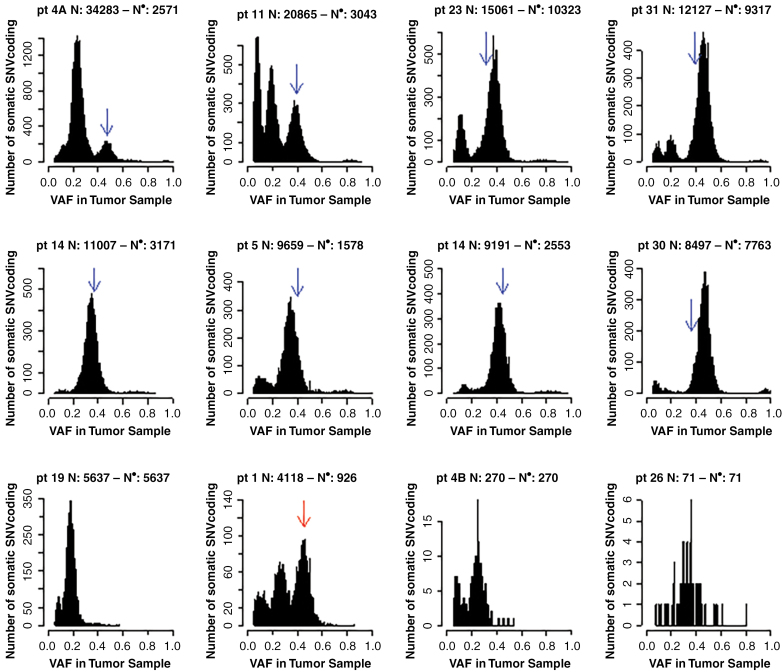
Mutation bursts in the 12 CMMRD patients. Histogram of the variant allele frequency (VAF) of somatic coding SNV for each patient. The VAF of the driving *POL* mutation is indicated with an arrow whenever present, blue for *POLE* and red for *POLD1*.

Analysis of the VAF distribution of the coding SNV revealed that there was more than one mutation burst in most of the tumors as reflected by distinct peaks in the histogram distribution (Figure 3). The largest one was not always the peak with the highest VAF. The driving *POLE* or *POLD1* mutation (respectively blue and red arrows in the panels of Figure 3) was always found in the first mutation burst, that is one with the highest VAF, but not always with the highest VAF within the burst (eg, patient 23, 30 and 31). Anyway, in all the cases the selected *POLE/POLD1* mutations were always associated with a VAF higher than 31% and more often around 40% as reported by Shlien et al. previously.^13^ Additional peaks contain mutations with lower VAF in all the 9 *POLE/POLD1* cases. These additional peaks present a smaller number of mutations, except in 3 patients (patients 4A, 11, and 1) where subsequent events had a larger contribution to the total mutation burden than the first peak).

### Mutation Spectrum of CMMRD-Associated Malignant Gliomas

The type of substitutions observed is globally homogenous among samples, G:C->A:T transitions (60% of the substitutions) and C:G-> A:T transversions (20% of the substitutions) being the most frequent (*data not shown*).

**Figure 4.**
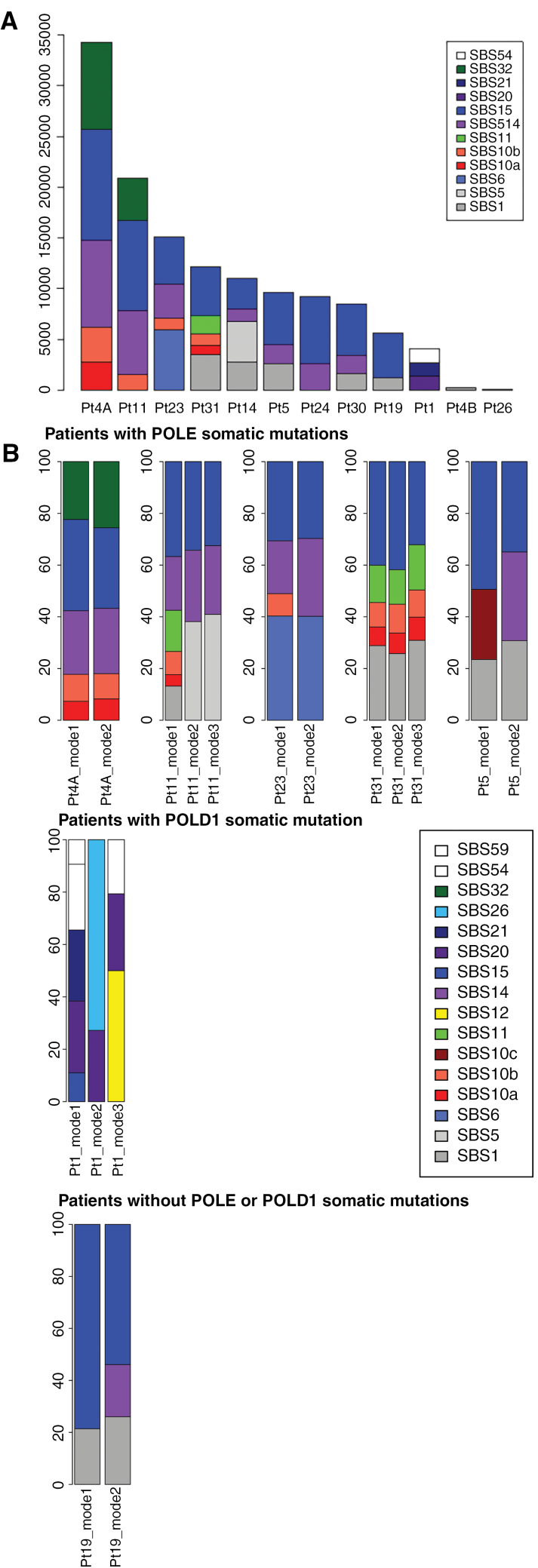
Mutational signature profiling with SigProfiler. (A) Mutational context of all the coding single nucleotide variations (SNV) according to the presence and type of *POLE/POLD1* mutation. (B) Mutational context of all the SNV according to each burst (mode) ordered by mean variant allele frequency (VAF) in patients with multiple bursts of mutations. The signature extraction is performed on each of the bursts independently. Signatures are extracted similarly to the description of Alexandrov et al., Nature 2020.

Then, we studied the mutational context in which the variations occurred to describe the mechanisms of the cancer development with respect to mutations generation. For each patient, we analyzed both the whole mutational spectrum and the distinct mutation bursts individually using SigProfiler, a supervised approach, to look for the presence of the 67 mutational signatures (as defined in the version v3.4 of October 2023: https://cancer.sanger.ac.uk/signatures/sbs/). The type of signatures identified were relatively homogeneous with predominance of signature SBS 15 (Defective DNA Mismatch Repair), SBS 14 (Concurrent Polymerase Epsilon Mutation and Defective DNA Mismatch Repair), and SBS 10a and 10b (Polymerase Epsilon Exonuclease Domain mutations) (Figure 4A). The signature SBS 14 was present in all samples where a driver *POLE* mutation could be identified but one (7/8), although in most of the cases this signature represented only a small proportion of the mutations. In patient 31, no signature 14 was found but instead 2 signatures 10a and 10b were observed. This finding, highlighted here, was also mentioned in the study of hypermutated tumors from the Toronto group.^14^ Two clock signatures SBS 1 and 5 were also observed in a few samples. Patient 1 had no *POLE* mutation-associated signature but only the signature SBS 20 (Concurrent *POLD1* mutation and Deficient DNA Mismatch Repair) compatible with the *POLD1* mutation in the multifunctional domain identified with a high VAF (45.3%) (Supplementary Table 4).

We then compared the signatures of different mutation bursts in a given patient, limiting our analysis to the 7 samples with large enough peaks for the computation: that is, peaks with >500 mutations and >5% of the total number of mutations. We showed that the distribution of the signatures did not markedly change in each of the distribution modes (patients 4A, 5, 11, 19, 23, 31) (Figure 4B). If we assume that the different modes correspond to different sequential bursts of mutations, those with lower VAFs supposedly occurring after the occurrence of the selected driver *POLE/POLD1* mutations, that is in the subsequent modes with lower VAF, are not enriched with mutations associated with the polymerase mutated signature. Therefore the *POLE* mutations do not alter massively the pattern of alterations occurring after it, that is, with lower VAFs. This new finding indicates that the mechanisms of mutation remain pretty stable overtime and is not deeply modified by the *POLE/POLD1* mutation.

Finally, joint inference of tumor-intra heterogeneity and mutational processes was performed using CloneSig. Between 1 and 5 clones were identified by patient, a limited number given the high number of mutations. For example, the patient 4A has around 35,000 somatic coding mutations but only 3 clones. Except one patient, all patients had one major clone and one or several minor clones. (Supplementary Figures 3 and 4). The signature activity of the different mutational processes acting in the tumors are very similar in the different clones (Supplementary Figure 4). This analysis indicates that POLE/POLD1 did not introduce clones with different mutational profiles and that, even though the mutation rate is very high, the mutation processes are not random but instead still well organized.

### Qualitative Analysis of the Mutations

On the contrary to the most frequent mutations observed in brain tumors in COSMIC or in pediatric HGG,^26^ no K27M mutation in histone H3 was observed. A R132H mutation in *IDH1* was identified in only one patient with low VAF. No *BRAF* V600E mutation was seen. Among the most frequently mutated genes in adult glioblastomas, no *EGFR*, *PTEN,* or *RB1* alterations were reported among the most frequently mutated genes in CMMRD-associated gliomas.

**Figure 5.**
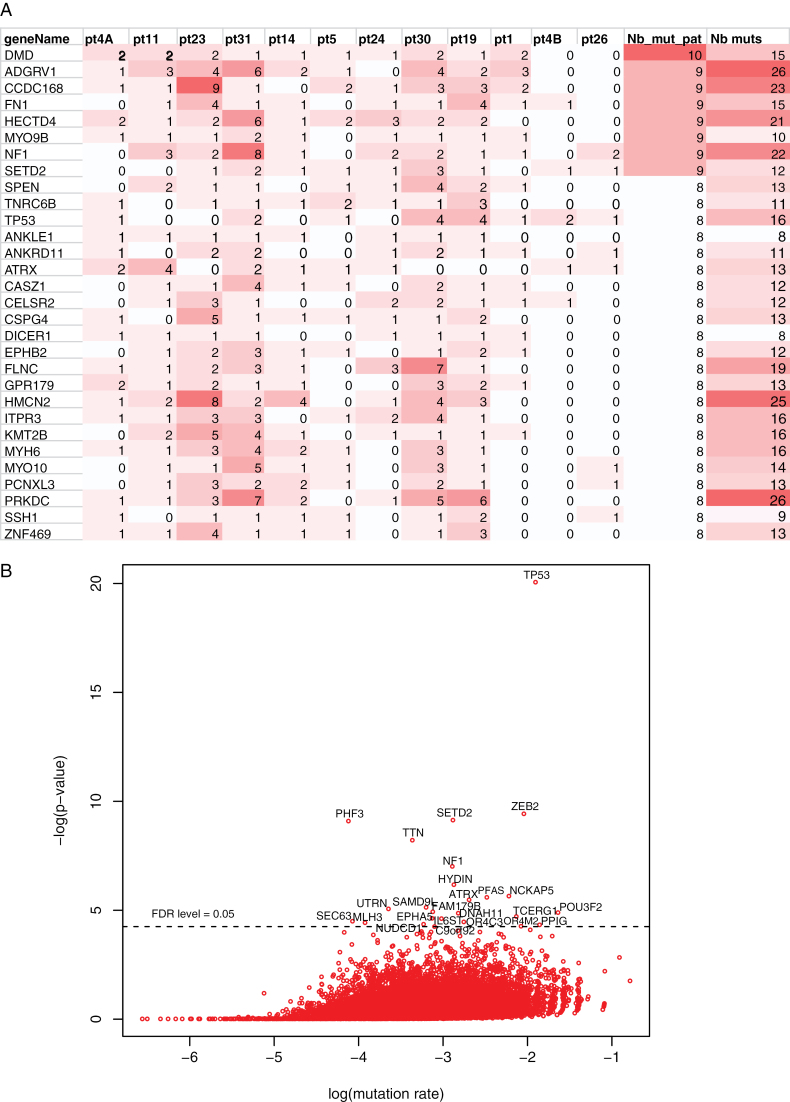
Most frequently mutated genes. (A) Genes with mutations (synonymous SNV excluded) with higher variant allele frequency (VAF) than the *POLE/POLD1* mutation in at least 8/12 (66%) of the samples are shown ordered by recurrence. Each column represents one patient. The heatmap is color coded according to the number of coding mutations in each given gene. Nb_mut_pat = number of patients with mutation in a given gene. Nb_muts = number of mutations in total in all patients for a given gene. (B) To identify the mutated genes with potential driver role in CMMRD-associated gliomas, DrGaP analysis was conducted on somatic coding mutations, including the synonymous mutations. The score on the y-axis compares for a given gene, the number of synonymous and non-synonymous mutations, the higher the score the more likely the gene plays a role in the oncogenesis.

Mutations with high VAF, that is, superior to the one of *POLE/POLD1*, can be considered either as occurring before or being selected during oncogenesis. They represent anyway important targets for potential therapies and we select them in each patient. For the patients without *POLE* or *POLD1* mutation, all their variants were considered. Synonymous SNV were excluded. We then selected the 30 most-frequently mutated genes with at least one coding somatic variant in at least 8 out 12 patients (Figure 5A). *TP53* mutations were present in 8/12 cases and usually with a high VAF close to 50%. However, no LOH was observed at the *TP53* locus in any of the samples. Four patients had more than one deleterious mutation in *TP53* indicating the possible existence of subclones and the evolutive pressure to acquire *TP53* mutations during oncogenesis of these tumors. The pressure to inactivate the p53 pathway is further emphasized by the occurrence of sub-clonal mutations in *PPM1D* (in 4 patients 11,19, 23 and 31) or LOH in *TP53BP1* (in patient 4B) as well as deleterious sub-clonal mutations in *TP53BP1* (in 6 patients 11, 23,14, 19, 30 and 31). Among DNA repair genes, the most frequently mutated was *PRKDC* (8 patients, 67%) again with multiple mutations in 5 samples out of the 8 mutated ones. *NF1* and *SETD2* were among the most frequently mutated genes both in 9 (75%) of the samples, pointing towards alternative oncogenic pathways compared to most common types of pediatric HGGs. Links between CMMRD and NF1 have already been mentioned and they may be more complex than previously thought.^27-30^ *NF1* somatic mutations are rarely found in pediatric HGGs outside CMMRD predisposition and *SETD2* mutations have been described in around 15% of supratentorial pediatric glioblastomas.^26^. Some of the frequently mutated genes have been rarely reported in brain tumors according to the COSMIC database: *EPH2B* (67% vs 1.11%), *DICER1* (67 vs 1.25%), *KMT2B* (67% vs 1.46%), *ATRX* (67% vs 13.95%) as well as several muscular genes (*DMD, MYO10, MYO9B, MYH6*) (*P* < .00001, Fischer exact test).

In addition, we investigated the potential “co-driver” among the genes frequently mutated (whatever their VAF) using DrGaP software, as shown in Figure 5B. For this analysis, we used all somatic coding SNV, including synonymous mutations. Among the mutated genes identified with potential co-driver role, several genes that could play a role in HGG development were identified: *TP53, NF1, ATRX, PRKDC, PHF3, SETD2, ZEB2, POU3F2.* In addition, 3 genes involved in ciliary dyskinesia were identified: *HYDIN*, *DNAH11*, and *FAM179B* as well as 2 olfactory receptor genes: *OR4M2* and *OR4C3*.

## Discussion

In this comprehensive study of CMMRD-associated HGGs, we show that their oncogenesis is more structured than anticipated and unique among other pediatric and adult glioblastomas. All the 9 patients with a driving *POLE* mutations are ultra-hypermutated (>100 coding SNV/Mb). Although they have a CMMRD molecularly confirmed, 3 of the 12 patients (patients 19, 4B, and 26) of this series did not have the required number of mutations to fulfill the criterion of ultra-hypermutated phenotype in their HGG. This indicates that this somatic diagnostic criterion although very useful must be completed with IHC staining or qualitative assessment of the mutational signature when this threshold is not reached.

Exploring more precisely the ultra-hypermutation phenomenon in this series of patients, we introduce some nuances in the mutational landscape previously described by Campbell and Shlien.^13,14^ Shlien showed that loss of function *POLE* mutation was associated with the ultra-hypermutation phenomenon.^13^ Then, Campbell considered all *POLE* mutations associated with ultra-hypermutation to be causative. We took a more conservative approach considering only the *POLE/POLD1* mutation disrupting the functional exonuclease domain. To characterize the impact of the selected *POLE/POLD1* driver mutation on the mutation load, we considered the mutations occurring before and after its occurrence, assuming that mutations with higher VAF than the one of the driver *POLE/POLD1* mutation would have occurred before it. We acknowledge that this assumption is acceptable only if there were no significant copy number aberrations in the *POLE/POLD1* genes (which is the case in this series of patients) and if we consider as in previous literature^13,14^ that there was no clone selection with specific mutations during oncogenesis.

These analyses led us to speculate that the ultra-hypermutation phenomenon may start before the occurrence of the polymerase driver mutation. Four patients had more than 100 mutations/Mb with higher VAF than the identified *POLE* driver mutation suggesting that ultra-hypermutation started before the occurrence of the *POLE* mutation or that these mutations were specifically selected. Mutations with lower VAF than the one of the driving polymerase (ie, supposedly occurring after the driving polymerase mutation) did not contribute to a significant increase of the mutation load as only one patient had an increase over 10-fold. In addition, although the VAF of the deleterious *POLE/POLD1* mutation was most often in the mutation burst with the highest VAF, its VAF was not necessarily the highest among the mutation burst.

When we identified the specific mutation signatures by mutational signatures deconvolution, we observed that the *POLE* mutation associated signatures were present in all but one sample harboring a loss of function *POLE* mutation (mostly signature SBS 14, seldomly signatures SBS 10a and 10b). Moreover, the tumor of patient 1 with a *POLD1* mutation had no *POLE* mutation associated signature but conversely the *POLD1* mutation associate signature. These findings could therefore be used for the search of the driving *POLE/POLD1* mutation. However, these signatures represented only a small proportion of the whole mutation burden which is in light with previous report.^14^ Most of the mutations were indeed associated with the signatures of mismatch DNA repair deficiency. It has been shown that defective *POLE* proofreading by itself was not sufficient to drive the ultra-hypermutation phenotype but needed the concomitant suppression of the DNA MMR.^31,32^ Interestingly, when analyzing patients with multiple mutation bursts, the signatures were similar in each burst. This suggests that the mechanism of mutation acquisition is similar throughout the cancer history irrespective of the *POLE/POLD1* mutation. We did not find however, possibly due to the small sample size, patient or tumor characteristic correlated with the TMB. For example, patient 4B was the older sister of patient 4A. Both had multifocal glioblastomas but only 4A showed the classical ultra-hypermutation phenotype, suggesting that it may not be a question of age at tumor onset nor MMR variant pathogenicity.

With respect to diagnostic features leading to the pathological suspicion of CMMRD-associated glioblastoma, the presence of giant cells may be an indicator of this diagnosis, especially because these features are rare in children.^11^ A previous report looking at giant cell glioblastoma found indeed that one out of ten of these tumors had MMR deficiency.^33^ And interestingly, these tumors have been shown to be more sensitive to DNA damage,^34^ which may be partly due to additional somatic alteration in DNA repair machinery.^13^ Moreover, alterations leading to p53 pathway dysfunction are very common in these CMMRD-associated HGGs suggesting that the hypermutation phenotype and alteration in the DNA repair machinery may trigger p53 activation from which the tumor has to escape for progression. This finding has not been described before in this setting. Whether these mutations could play a role in the resistance of these gliomas to therapy needs to be further explored. In addition to *TP53*, other genes involved in the DNA repair machinery were found mutated at a high frequency and associated with a VAF higher than the one of *POLE*, including *PRKDC*. In this respect, *PRKDC* mutations (67% in this series and in the report from Shlien^13^) were highly associated with MSI-high status,^35^ high tumor mutation burden, and inflamed tumor microenvironment.^36,37^ In our small series, there was no correlation between the presence of *PRKDC* mutations and T-cell immune infiltrates nor PD1 expression (*data not shown*). In addition, like other alterations triggering DNA reparation, these mutations may participated in the activation of specific immunogenic pathways.^38^ These early *PRKDC* alterations may participate in the oncogenesis through increased DNA damage^35^ in the gradual increase of mutations seen in CMMRD glioma before the onset of the *POLE/POLD1* mutation. Further studies are needed to identify the events that trigger the mutation bursts aside of *POLE/POLD1* mutations.

Our analyses of mutations with VAF higher than the one of the *POLE/POLD1* mutations and of potential driver genes point towards alterations that could represent new targets whether they occur early during oncogenesis or are selected during the evolution of the tumor.

MAPK pathway activation with frequent somatic *NF1* mutations, has been described in the majority of our patients, confirming the importance of this pathway in CMMRD biology where *NF1* mutations have been recently shown as mosaicism in some patients.^30^ Recent data from the Toronto group have explored the driver role of Ras/MAPK alterations in patients with MMR-deficient tumors and suggested a therapeutic efficacy of MEK inhibitors in selected patients.^29^ Targeted therapies against the MAPK pathway may therefore be of interest in conjunction with immune checkpoint inhibitors already explored in CMMRD patients.^10,39^

Our data also suggest areas to improve immunotherapy. *PRKDC* mutations are considered as both a biomarker for the response to PD1 inhibitors^35,36^ and a potential target to enhance the therapeutic response to PD1 inhibition. DNA-PK deficiency as a consequence of inactivating *PRKDC* mutations may also be another way to activate the c-GAS/STING pathway^37^ responsible for the strong immunogenicity of MMR associated tumors, in addition to the recently reported Exonuclease 1 hyperactivity.^37^ Therapeutic inhibition of DNA-PK may therefore be an interesting combination with PD1 inhibition in CMMRD-associated tumors without *PRKDC* mutations.^35^

In conclusion, our work shows an interest in exome sequencing of CMMRD-associated gliomas to better understand oncogenesis, complete the diagnostic work-out, and provide theranostic clues to define combination therapies unique to this category of gliomas. The ultra-hypermutation phenotype is associated with *POLE* or *POLD1* mutation but could start earlier through a different mechanism; in this respect, it could be hypothesized that the role of the *POLE* somatic mutation is instrumental for the introduction of *POLE*-associated mutations as well as permissive to MMR generated mutations. Other mechanisms leading to mutation accumulation should be considered like the *PRKDC* somatic mutations that could suggest interesting new therapeutic strategies.

## Supplementary Material

vdae120_suppl_Supplementary_Data

## Data Availability

Access to the raw data will be offered upon request.

## Notes

**Previous presentation:** This work was the subject of an oral presentation at the International Symposium of Pediatric Neuro-Oncology in Hamburg, June 13th, 2022.
