# Supplementary material for: Glioma oncogenesis in the Constitutional mismatch repair deficiency (CMMRD) syndrome
Source: Neurooncol Adv. 2024 Jul 11;6(1):vdae120. doi: 10.1093/noajnl/vdae120 (PMC11372297; doi:10.1093/noajnl/vdae120)
Supplement: vdae120_suppl_Supplementary_Data [file vdae120_suppl_Supplementary_Data.zip › suppl/Supplementary data_CMMRD_v11.docx]

**Supplementary Figures and Tables**

**Guerrini-Rousseau L & Merlevede J et al,**

**Supplementary Figure S1:**


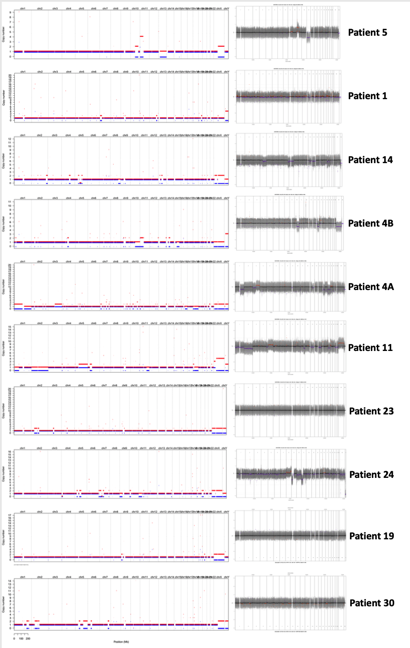


**Copy number variation (CNV) in CMMRD-associated gliomas**

Two different methods were used to study copy number variation in the exomes: Sequenza (left panel) and EaCoN (right panel). Copy number variation in tumor *versus* control blood sample is shown according to chromosomal location. Copy number is represented for each allele in red and blue in the left panel. Genomic regions presenting significant copy-number changes are shown in Orange (amplification) and purple (deletion) in the right panel. CNV profiles were mostly flat with few copy number changes.

**Supplementary Table S1: Patient’s medical history**

| **Previously described**  (Guerrini-Rousseau et *al.* NOA 2019) | **MMR**  **Mutated gene** | **First tumor**  **(age at onset)** | **Second tumor (age at onset)** | **Other tumors**  **(age at onset)** | **Status and age at last FU** |
| --- | --- | --- | --- | --- | --- |
| 1 | *PMS2* | **HGG** (5y) |  |  | Dead, 6.1y |
| 4. A | *MSH6* | **HGG** (11y) |  |  | Dead, 11.7y |
| 4.B | *MSH6* | **HGG** (13y) |  |  | Dead, 13.2y |
| 5 | *PMS2* | HGG (6y) | **HGG** (7y) |  | Dead, 8.7y |
| 11 | *MSH6* | TLL (7y) | TLL (11y) | **HGG** (14y), ADK (14y) | Dead, 16y |
| 14 | *PMS2* | **HGG** (6y) |  |  | Dead, 7.5y |
| 19 | *PMS2* | TLL (4y) | **HGG** (8y) |  | Alive in SD, 13.8y |
| 23 | *MSH6* | **HGG** (3y) |  |  | Dead, 5.7y |
| 24 | *PMS2* | **HGG** (7y) |  |  | Dead, 7.8y |
| 26 | *PMS2* | BLL (12y) | **HGG** (13y) |  | Dead, 13.3y |
| 30 | *MSH6* | HGG (6y) | **HGG** (6y) |  | Dead, 6.9y |
| 31 | *PMS2* | **HGG** (10y) | HGG (13y) | TLL (13y) | Alive in CR, 13.9y |

HGG: High-grade glioma, TLL/BLL: T/B lymphoblastic lymphoma, ADK: digestive Adenocarcinoma, FU: follow-up, y: years, SD: stable disease, CR: complete remission.

The HGG analysed in the study are highlighted in bold in case the patient had more than one HGG during follow-up.

**Supplementary Table S2: Mutations and functional tests**

| **Patient ID** | **Gene** | **Mutation(s)** | **Variant classification and ACMG criteria** | **Functional testing** |
| --- | --- | --- | --- | --- |
| 1 | *PMS2* | c.[2007-2A>G];[2007-2A>G],  p.[?];[?] * | Pathogenic (PVS1, PM2, PM3, PP4) | Abnormal  (MT and evMSI tests) |
| 4. A | *MSH6* | c.[3725G>A];[3725G>A],  p.[(Arg1242His)];[(Arg1242His)] | Likely Pathogenic (PM2, PM3, PP3, PP4) | Abnormal  (MT and evMSI tests) |
| 4.B | *MSH6* | c.[3725G>A];[3725G>A],  p.[(Arg1242His)];[(Arg1242His)] | Likely Pathogenic (PM2, PM3, PP3, PP4) | Abnormal  (MT and evMSI tests) |
| 5 | *PMS2* | c.[2007-2A>G];[2007-2A>G],  p.[?];[?] * | Pathogenic (PVS1, PM2, PM3, PP4) | NA |
| 11 | *MSH6* | c.1763_1771dup(;)(1763_1771dup),  p.(His588_Pro590dup)(;) (His588_Pro590dup) | Uncertain significance (PM2, PM4, PP4) | Abnormal  (MT and evMSI tests) |
| 14 | *PMS2* | c.[2007-2A>G];[2007-2A>G], p.[?];[?] * | Pathogenic (PVS1, PM2, PM3, PP4) | Abnormal (MT, gMSI and evMSI tests) |
| 19 | *PMS2* | c.[2007-2A>G];[2007-2A>G], p.[?];[?] * | Pathogenic (PVS1, PM2, PM3, PP4) | Abnormal (MT, gMSI and evMSI tests) |
| 23 | *MSH6* | c.[3386_3388del];[3386_3388del], p.[(Cys1129_Val1130delinsLeu)];  [(Cys1129_Val1130delinsLeu)] | Likely Pathogenic (PM2 + PM3 + PM4 + PP4) | gMSI normal |
| 24 | *PMS2* | c.[(705+1_706-1)_(803+1_804-1)del]; [(705+1_706-1)_(803+1_804-1)del], p.[(Leu236Hisfs*30)];[(Leu236Hisfs*30)] | Pathogenic (PVS1, PM2, PM3, PP4) | Abnormal  (MT and evMSI tests) |
| 26 | *PMS2* | c.[(2275+1_2276-1)_(*160_?)del];[803+2T>G],  p.[?];[?] | Pathogenic (PVS1, PM2, PM3, PP4) | Abnormal  (MT and evMSI tests) |
| 30 | *MSH6* | c.1800_1813dup(;)(1800_1813dup), p.(Thr605Ilefs*10)(;)(Thr605Ilefs*10) | Likely Pathogenic (PVS1, PM2) | NA |
| 31 | *PMS2* | c.[634C>T];[c.1239del], p.[(Gln212*)];[(Asp414Thrfs*34)] | Pathogenic (PVS1, PM2, PM3, PP4) | Abnormal  (MT and evMSI tests) |

Mutations in MMR genes are indicated following HGVS nomenclature for both cDNA and protein sequences. The mutations are classified according to the impact on the protein: deleterious variant (DV) for class 5 and class 4 variants, variant of unknown significance (VUS), for class 3 variants. Functional test performed: gMSI (genomic microsatellite instability) (S25), evMSI (*ex vivo* microsatellite instability) (S26) and methylation tolerance (MT) test (resistance of lymphocytes to alkylating agents like temozolomide) (S27). NA, not available.

* Same *PMS2* Pathogenic Variant for patients 1, 5, 14 and 19. All from Tunisia origin.

**Supplementary Table S3: Mutation counts in tumors**

| **Patient** | **Nb of all somatic SNV** | **Nb of all somatic SNV/Mb** | **Nb of coding SNV** | **Nb of coding SNV/Mb** | **Nb of coding SNV before POLE/POLD1** | **Nb of coding SNV/Mb before POLE/POLD1** |
| --- | --- | --- | --- | --- | --- | --- |
| 4.A | 69576 | 1288.4 | 34283 | 634.9 | 2571 | 47.6 |
| 11 | 46597 | 862.9 | 20865 | 386.4 | 3043 | 56.3 |
| 23 | 30257 | 560.3 | 15061 | 278.9 | 10323 | 191.2 |
| 31 | 25463 | 471.5 | 12127 | 224.6 | 9317 | 172.5 |
| 14 | 22760 | 421.5 | 11007 | 203.8 | 3171 | 58.7 |
| 5 | 18946 | 350.9 | 9659 | 178.9 | 1578 | 29.2 |
| 24 | 16633 | 308.0 | 9191 | 170.2 | 2553 | 47.3 |
| 30 | 18934 | 350.6 | 8497 | 157.3 | 7763 | 143.8 |
| 19 | 8687 | 160.9 | 5637 | 104.4 | 5637 | 104.4 |
| 1 | 9872 | 182.8 | 4118 | 76.3 | 926 | 17.1 |
| 4.B | 475 | 8.8 | 270 | 5 | 270 | 5 |
| 26 | 156 | 2.9 | 71 | 1.3 | 71 | 1.3 |
| **Median** | **18940** | **350.7** | **9425** | **174 .5** | **2807** | **52.0** |
| Minimum | 156 | 2.9 | 71 | 1.3 | 71 | 1.3 |
| Maximum | 69576 | 1288.4 | 34283 | 634.9 | 10323 | 191.2 |

**Supplementary Table S4: POLE and POLD1 secondary driver mutations identified**

| **Patient** | **Number of coding SNV/Mb** | **Mutated MMR Gene** | **Somatic *POLE* variant** | **Somatic *POLD1* variant** | **VAF** | **Comments *POLE*** |
| --- | --- | --- | --- | --- | --- | --- |
| 4.A | 1120.4 | *MSH6* | c.1381T>C, p.(Ser461Pro) |  | 47.2 | Well-established driver mutation |
| 11 | 681.9 | *MSH6* | c.1376C>T, p.(Ser459Phe) |  | 39.3 | Well-established driver mutation |
| 23 | 492.2 | *MSH6* | c.1231G>T, p.(Val411Leu) |  | 31.5 | Well-established driver mutation |
| 31 | 396.3 | *PMS2* | c.1376C>A,  p.(Ser459Tyr) |  | 39.3 | Novel missense change at an amino acid residue where a different missense well-established driver mutation is known |
| 14 | 359.7 | *PMS2* | c.3229C>T. p.(Arg1077Cys) |  | 37 | VUS |
| 5 | 315.7 | *PMS2* | c.1376C>A. p.(Ser459Tyr) |  | 40 | Novel missense change at an amino acid residue where a different missense well-established driver mutation is known |
| 24 | 300.4 | *PMS2* | c.830A>G. p.(Glu277Gly) |  | 44.8 | Missense change at an conserved carboxylate amino acid residue  in the ExoI domain |
| 30 | 277.7 | *MSH6* | c.2933A>G. p.(Glu978Gly) |  | 36.3 | Established driver mutation by Campbell *et al*. 2017 |
| 19 | 184.2 | *PMS2* |  |  |  |  |
| 1 | 134.6 | *PMS2* |  | c.2630A>G, p.(Asp877Gly) | 45.3 | VUS |
| 4. B | 8.8 | *MSH6* |  |  |  |  |
| 26 | 2.3 | *PMS2* |  |  |  |  |

VUS: variant of uncertain significance

**Supplementary Figure S2: Mutational spectrum for each patient derived from coding SNVs**

Each sample plot represents the mutational spectrum of a patient. It shows which mutational signatures were identified using SigProfilerAssignment and their contribution. Then, each profile is reconstructed using the underlying processes and their contribution. Finally, different statistics comparing the initial profile to the reconstructed one are provided.


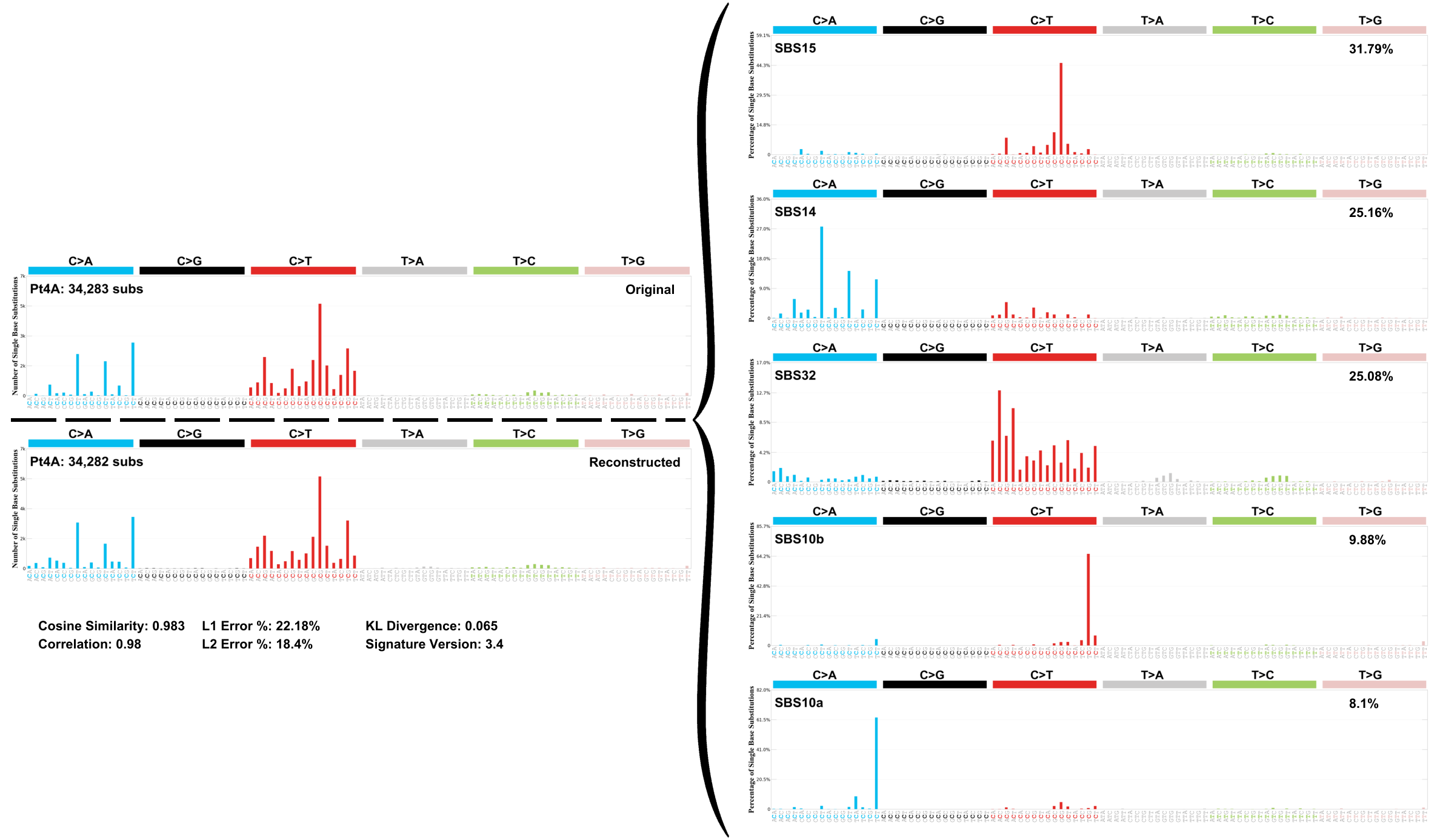


**
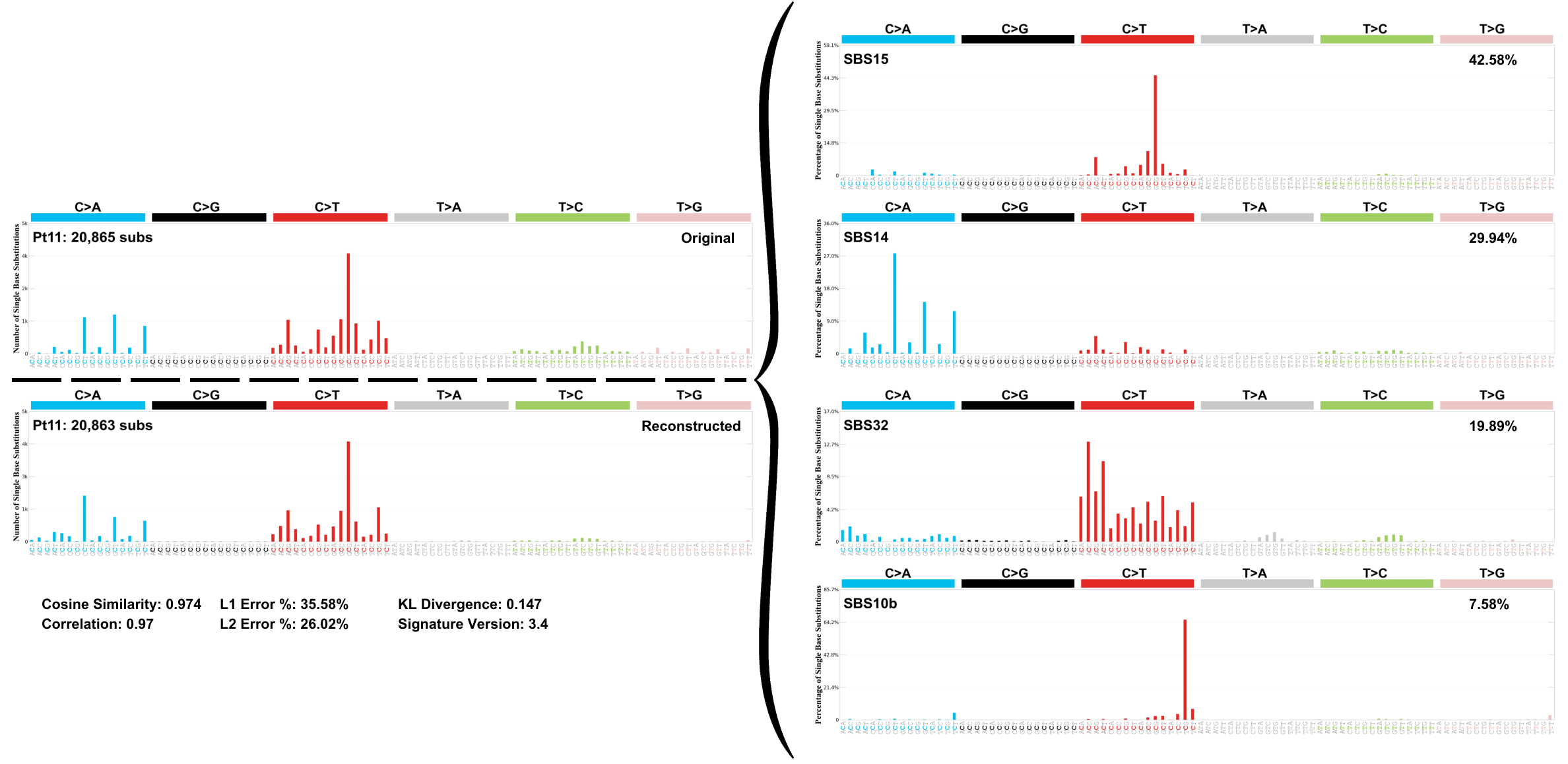
**

**
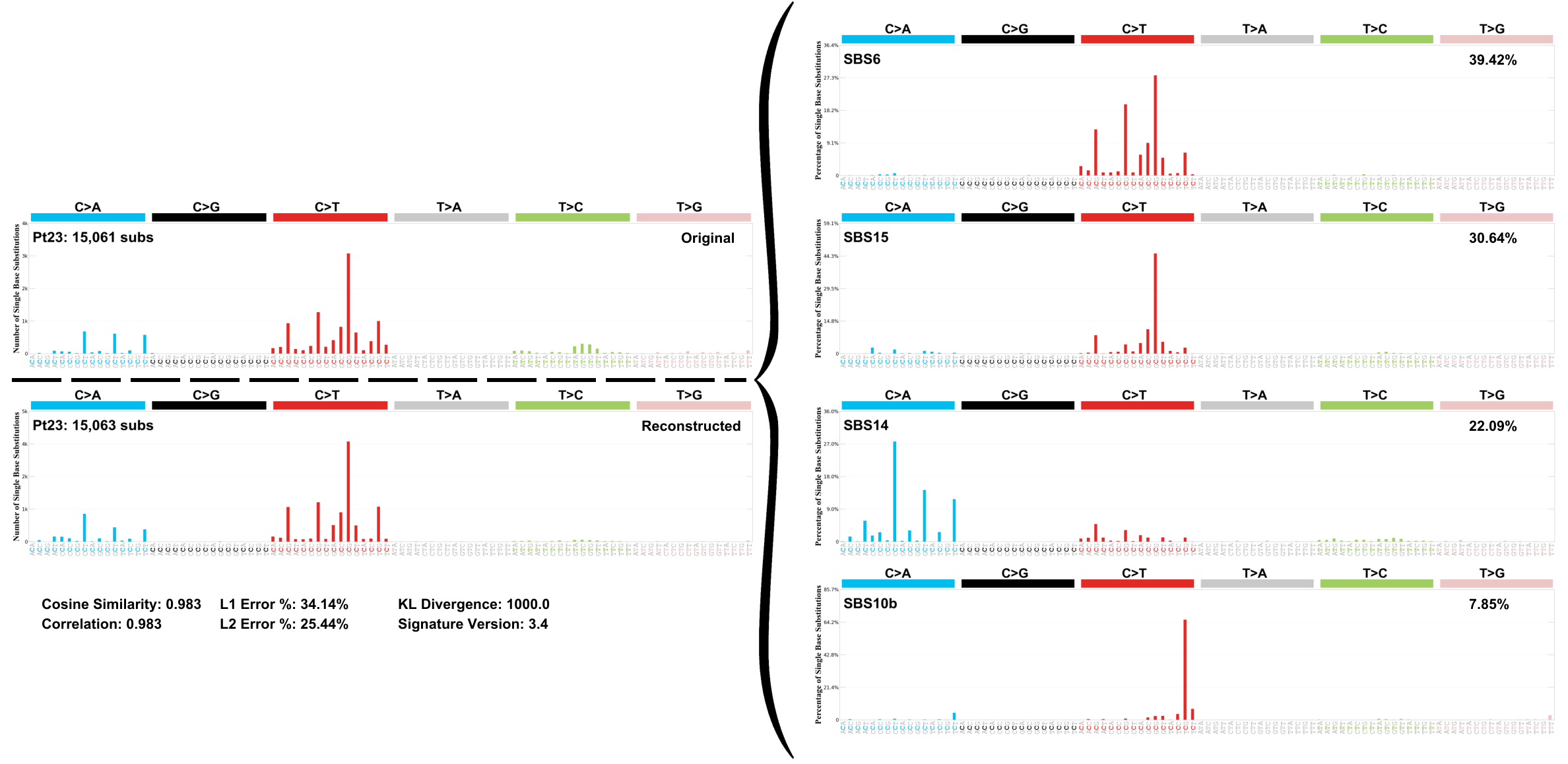
**

**
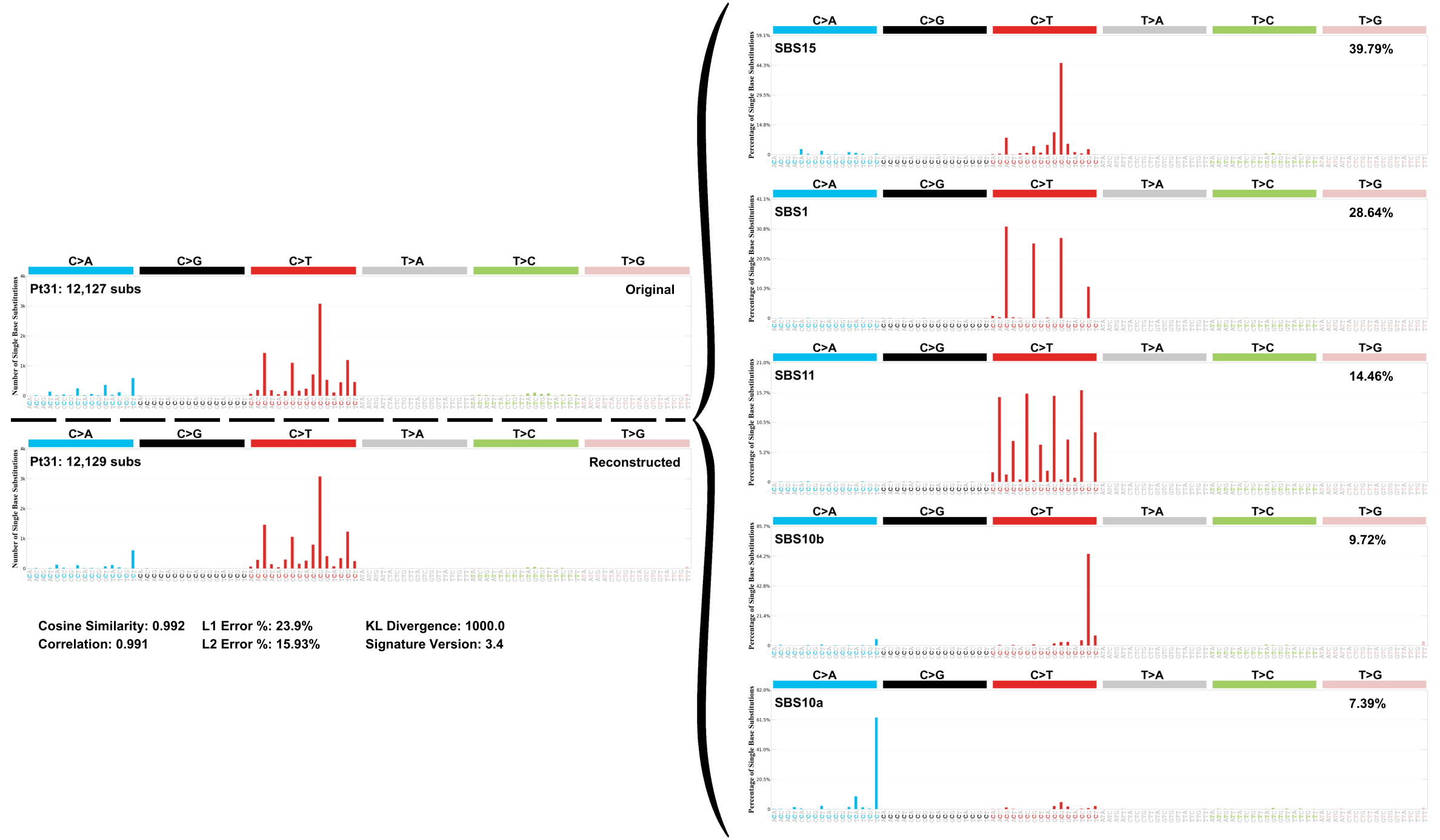
**

**
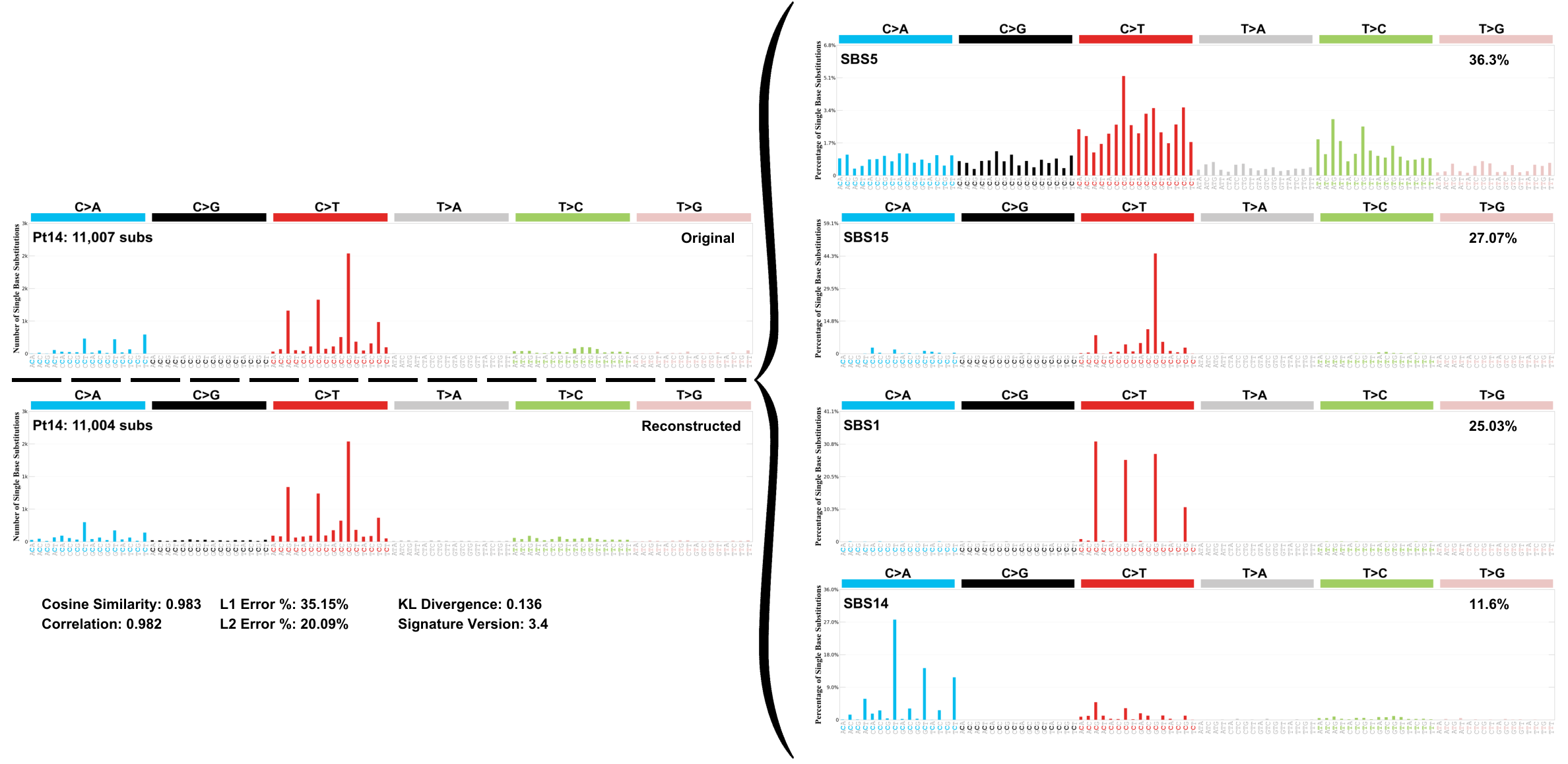
**

**
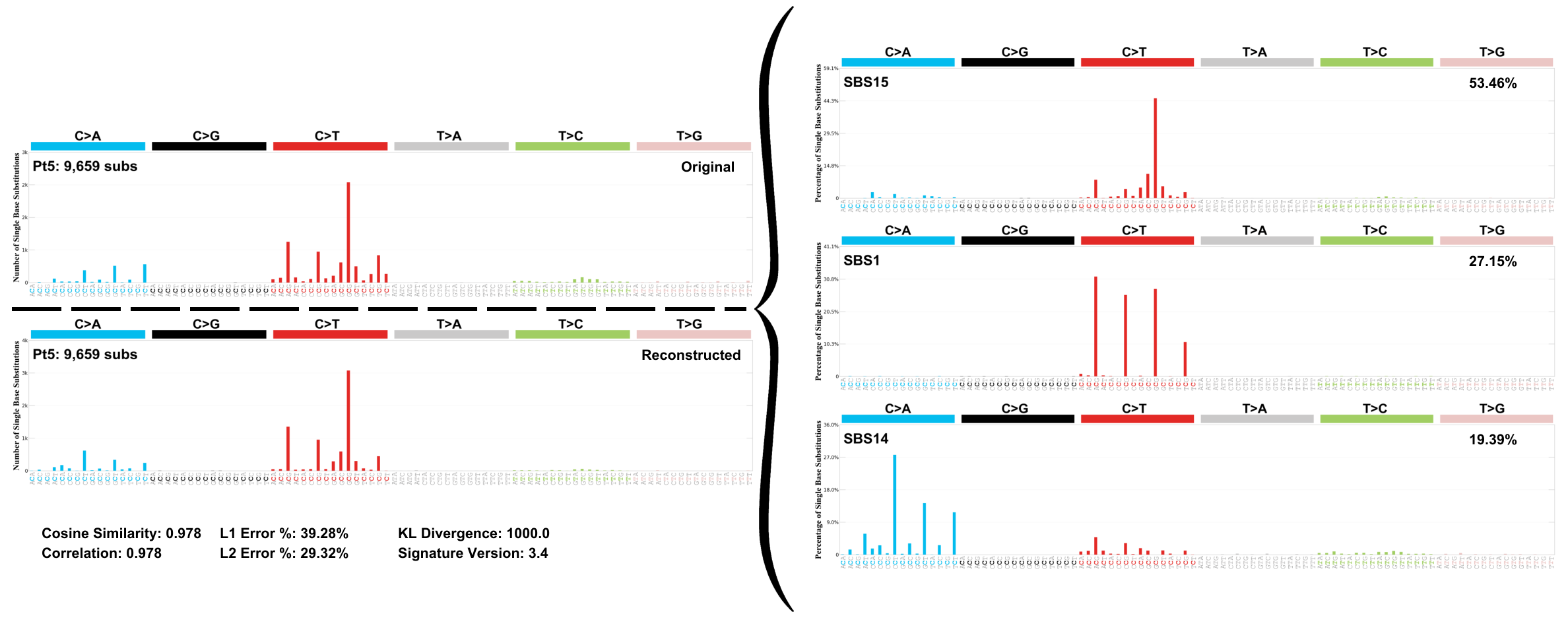
**

**
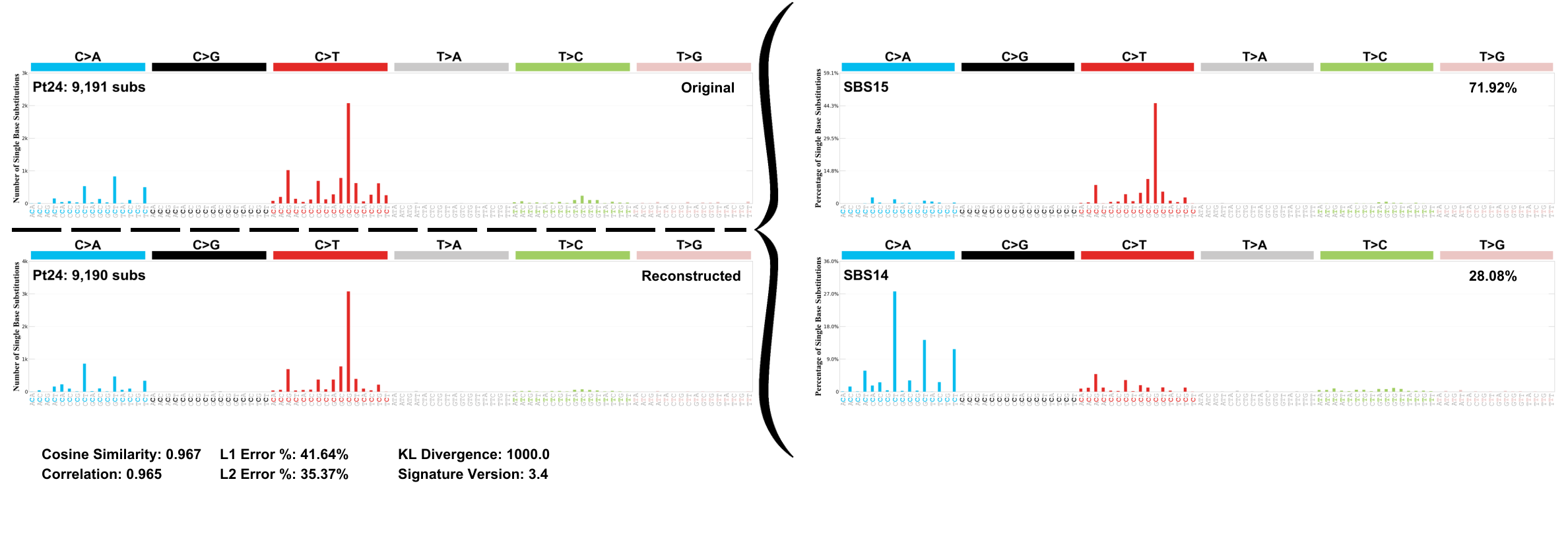
**

**
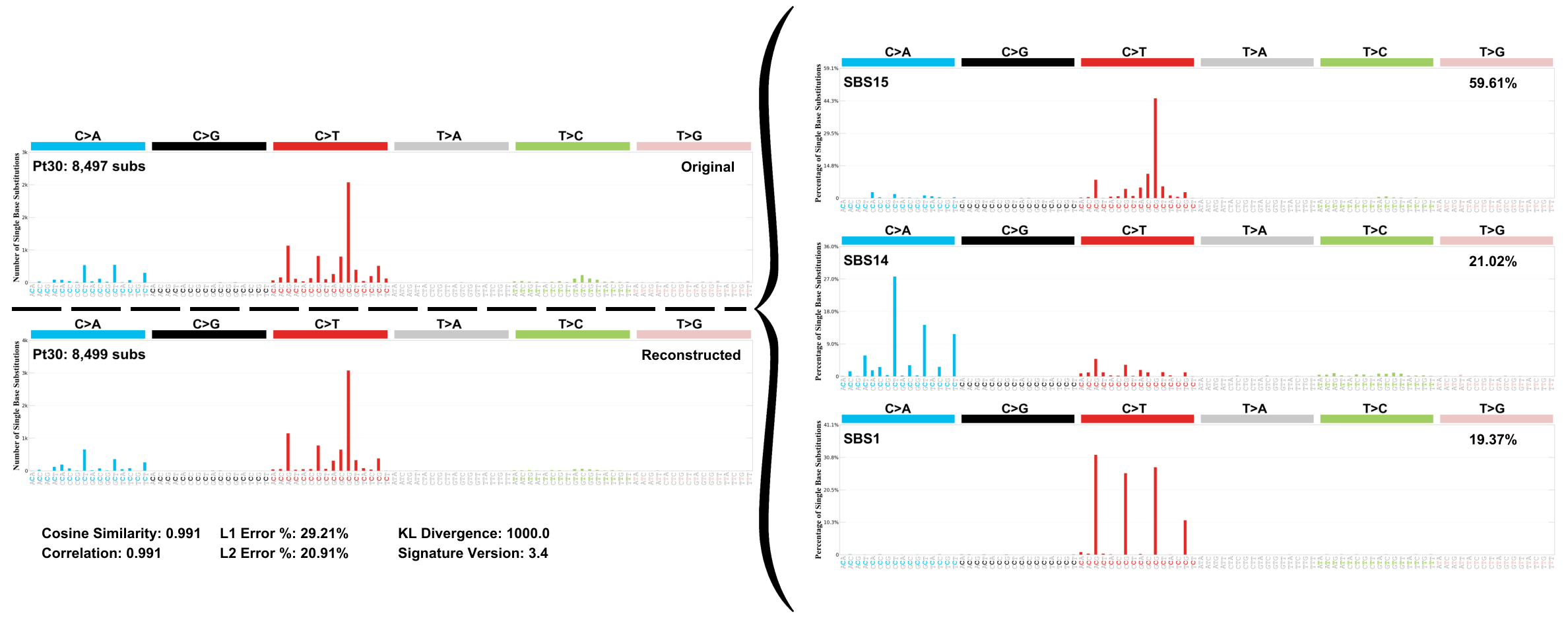
**

**
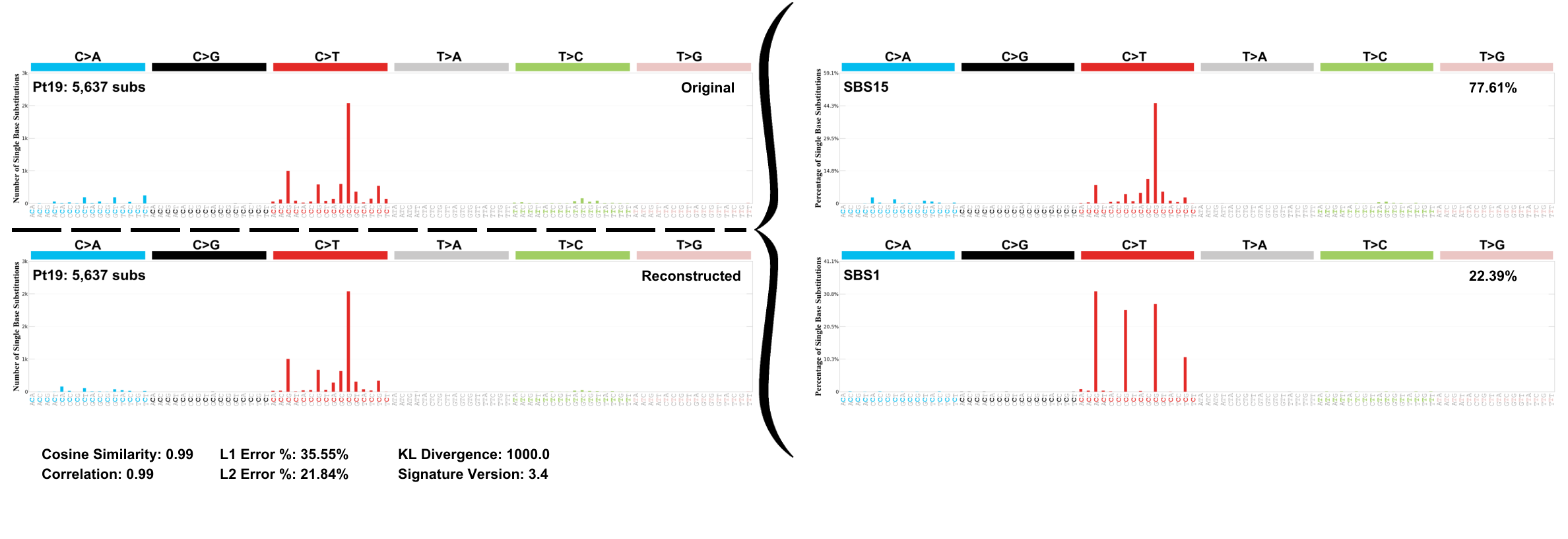
**

**
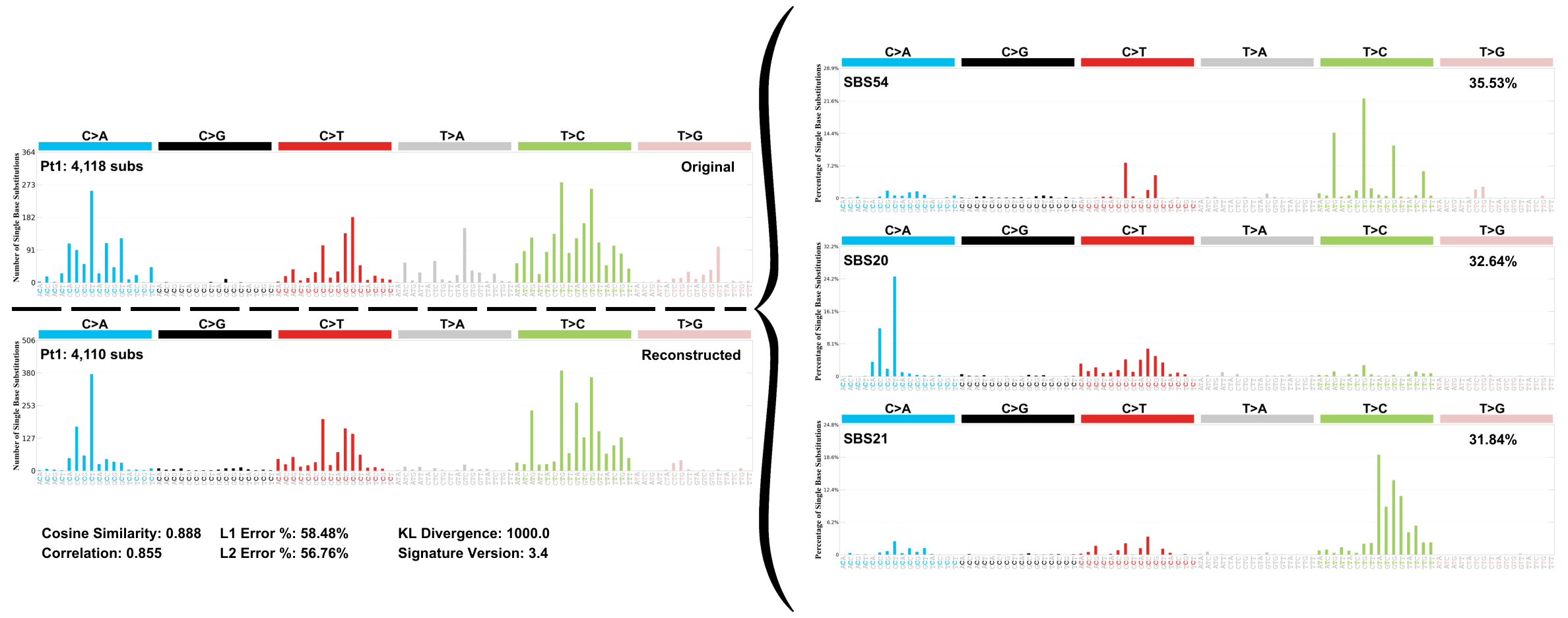
**

**
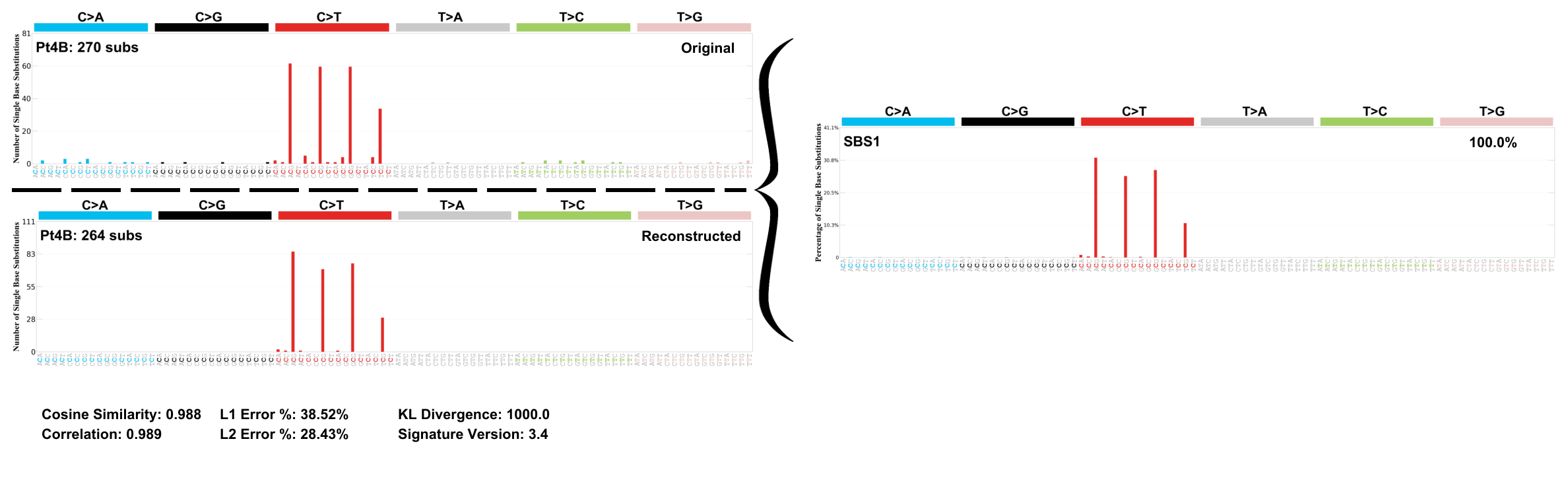
**


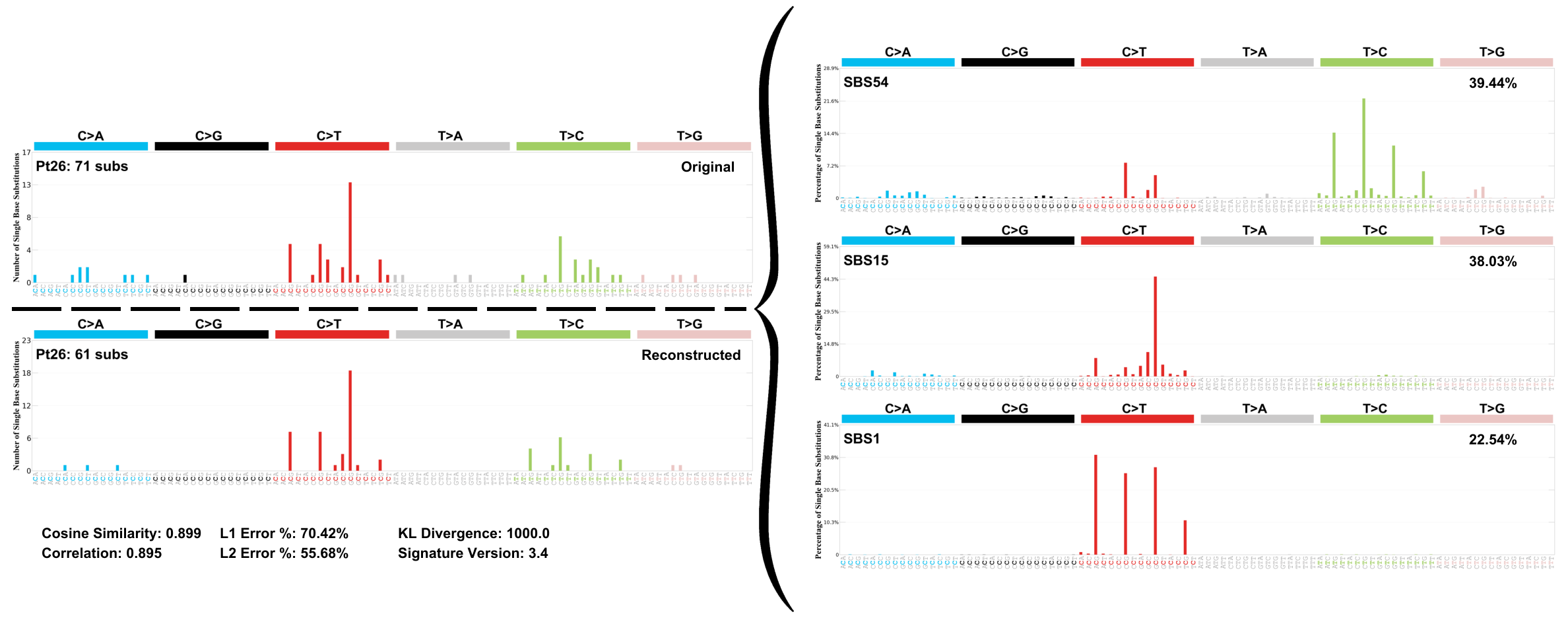


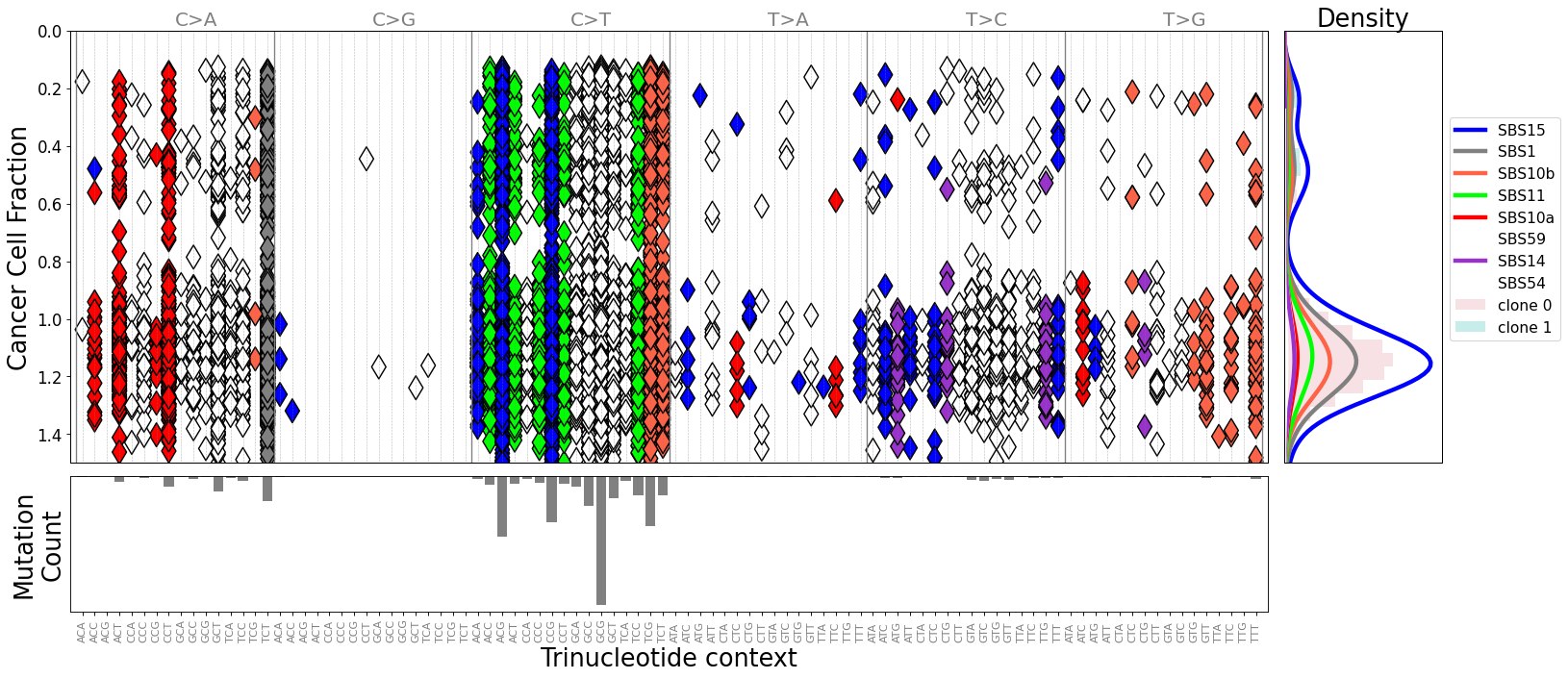
**Supplementary Figure S3: Jointly inference of intra-tumor heterogeneity and mutational processes for each patient**

For each patient, the main panel displays the somatic coding SNVs in 2 dimensions: horizontally the mutation type, which describes the type of substitution together with the flanking nucleotides, and vertically the estimated cancer cell fraction computed by CloneSig.

From these data, CloneSig infers the number of clones and the number of mutational signatures active in the different clones. Each mutation in the main panel is colored according to the most likely mutational signature it is from according to CloneSig. On the right panel, the cancer cell fraction histogram is represented and colored with estimated clones, and superimposed with mutational signature density. The bottom panel represents the total mutation type profile.


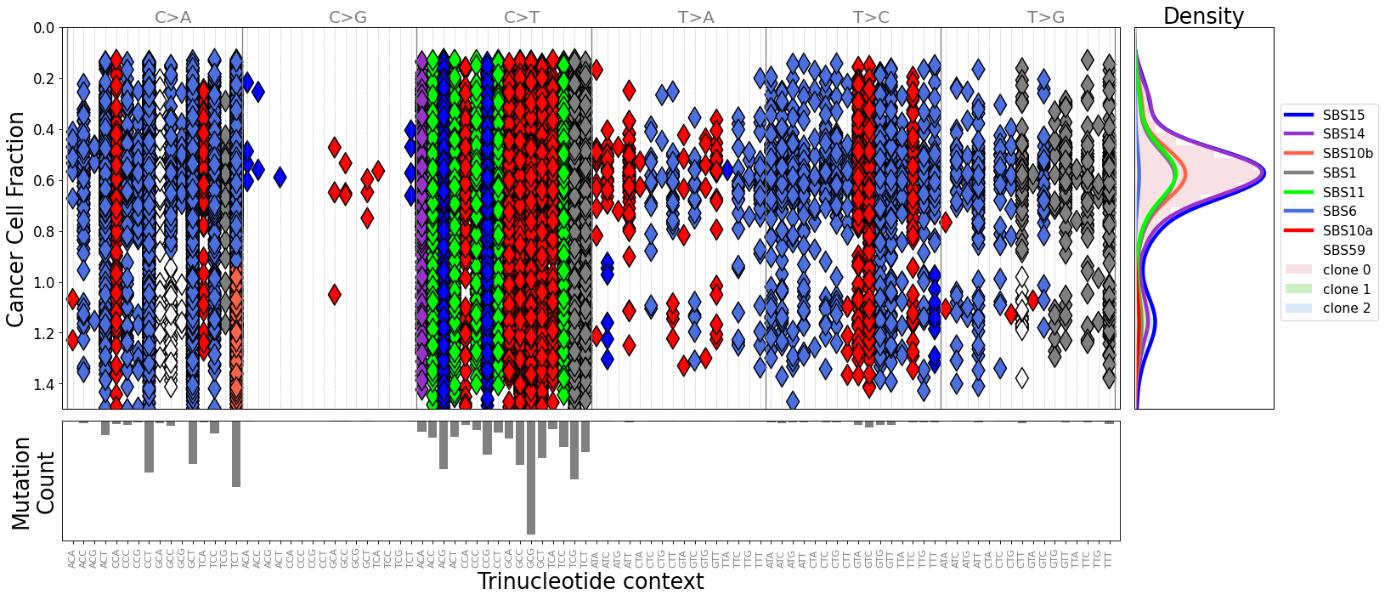


Pt4A


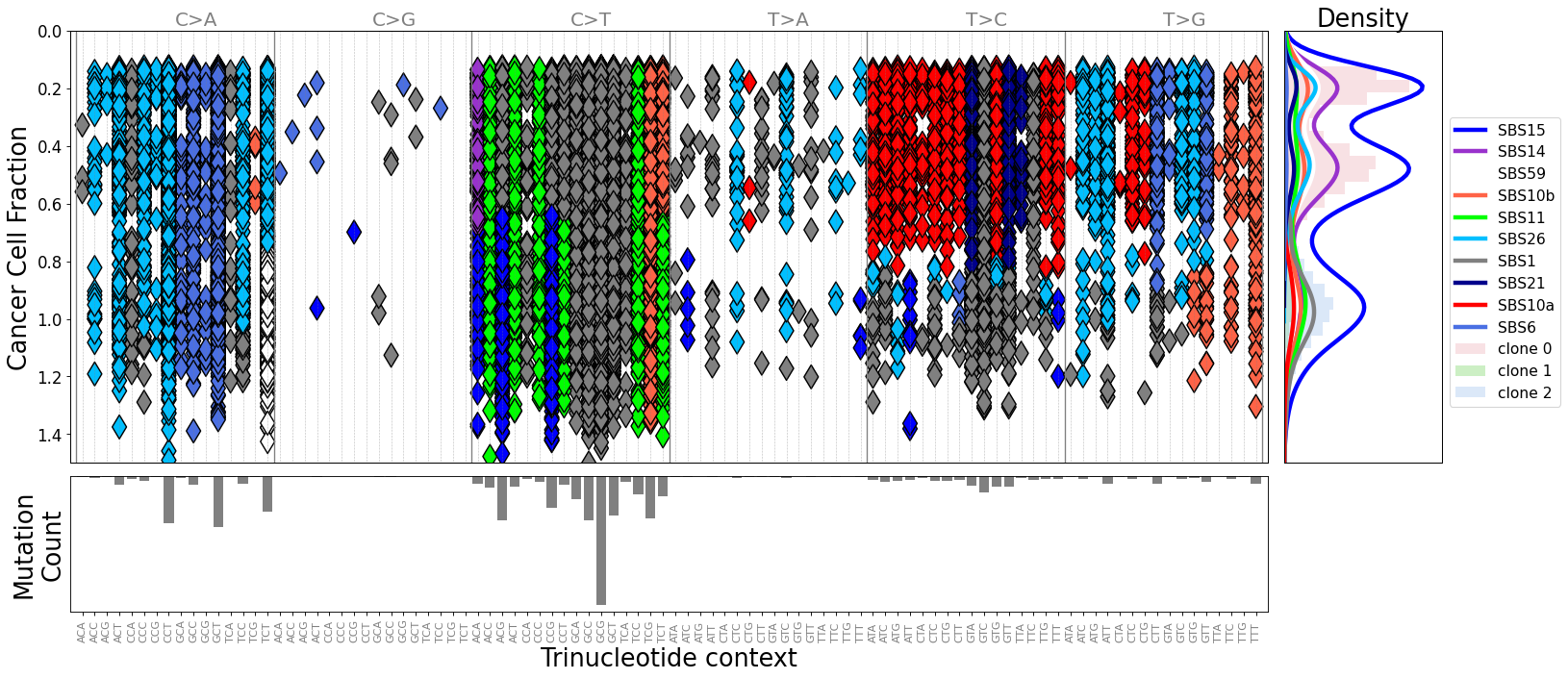


Pt11

**
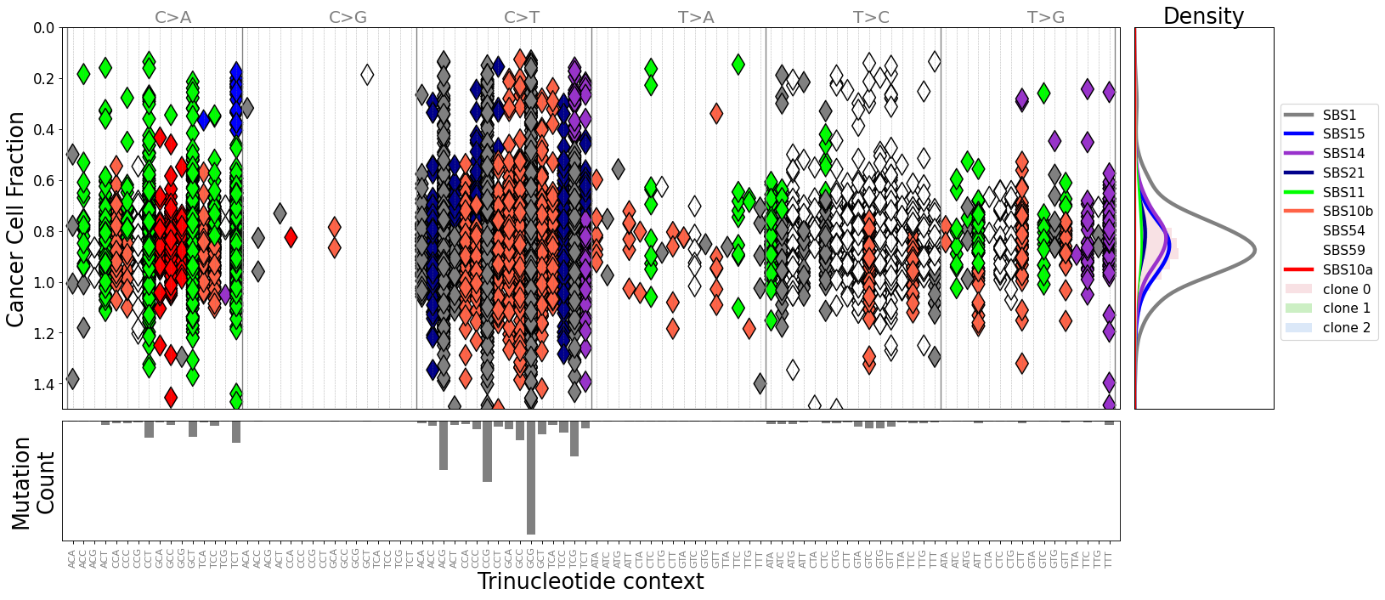
**


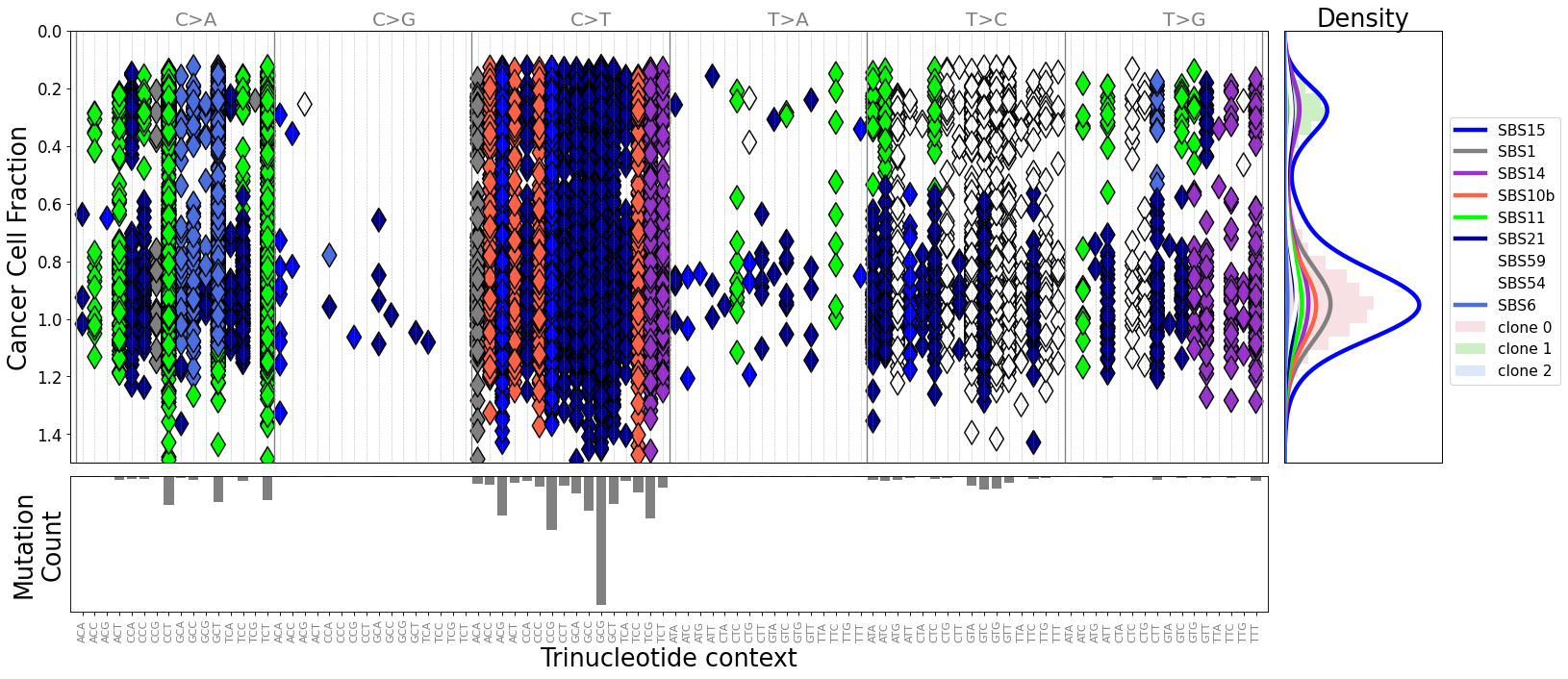


Pt23

Pt31


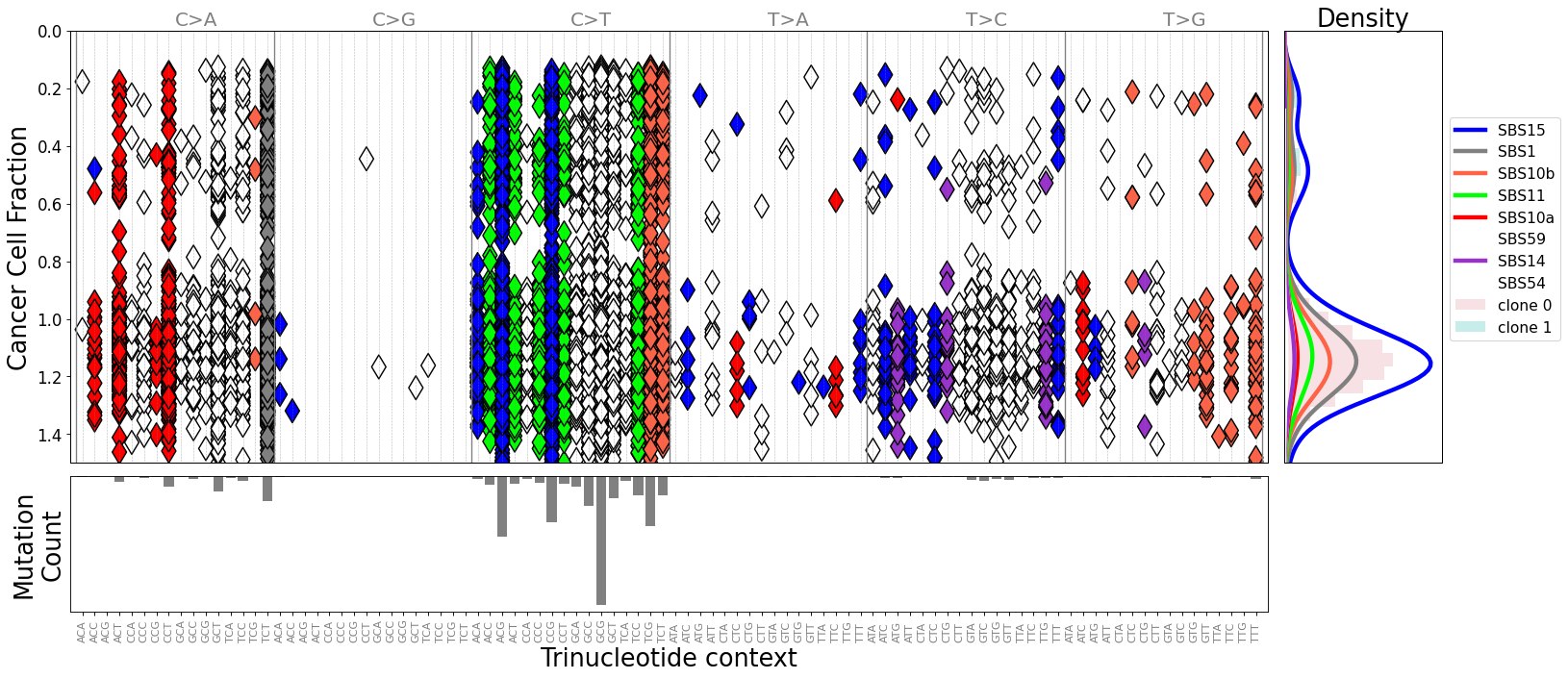

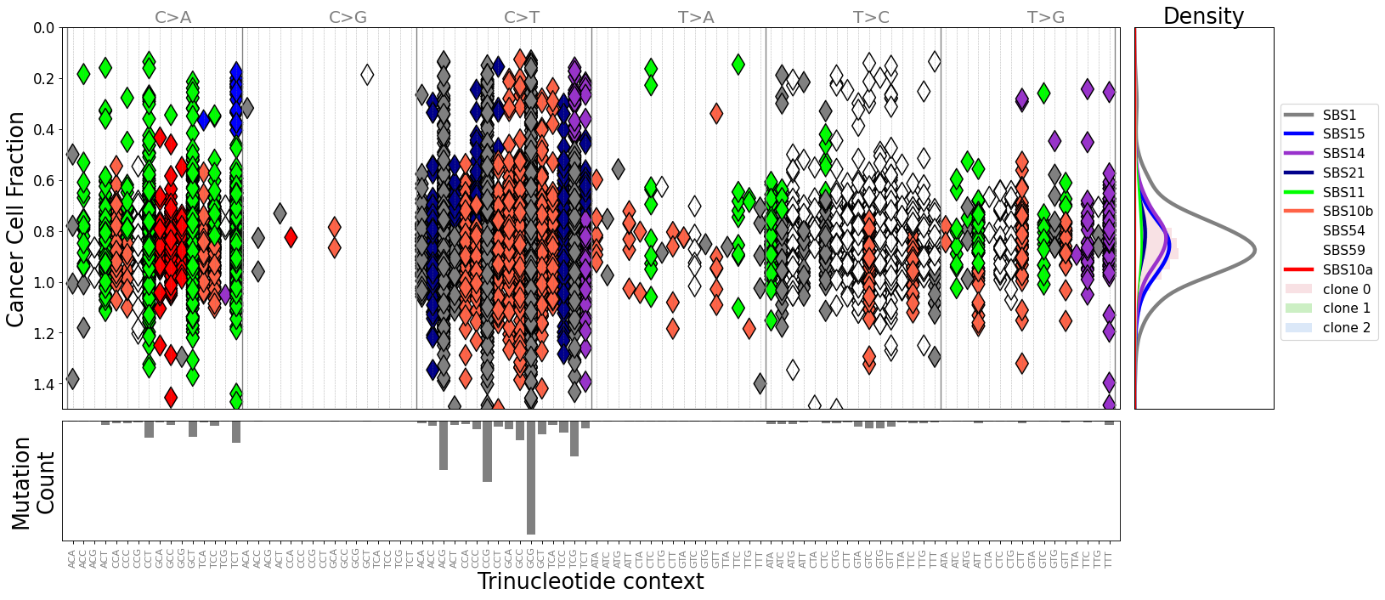


Pt14


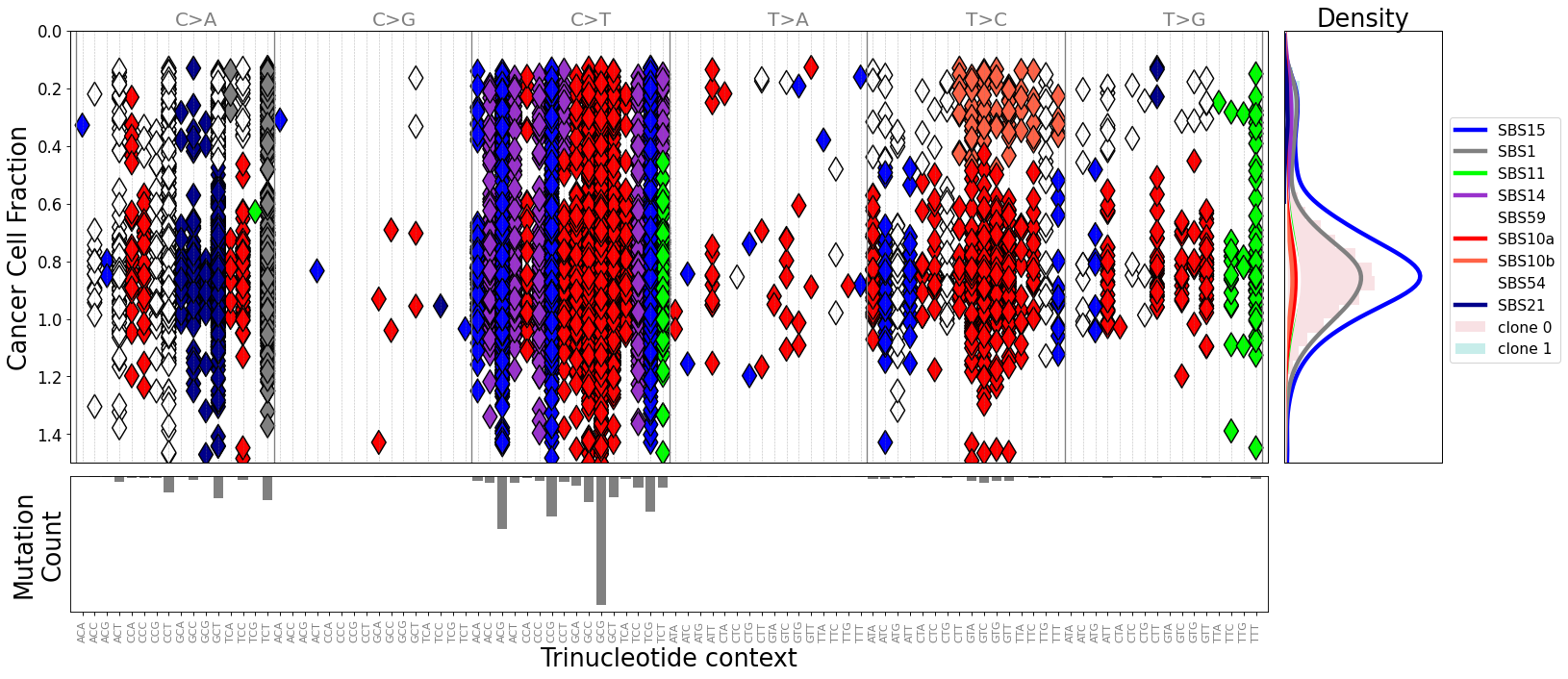


Pt5


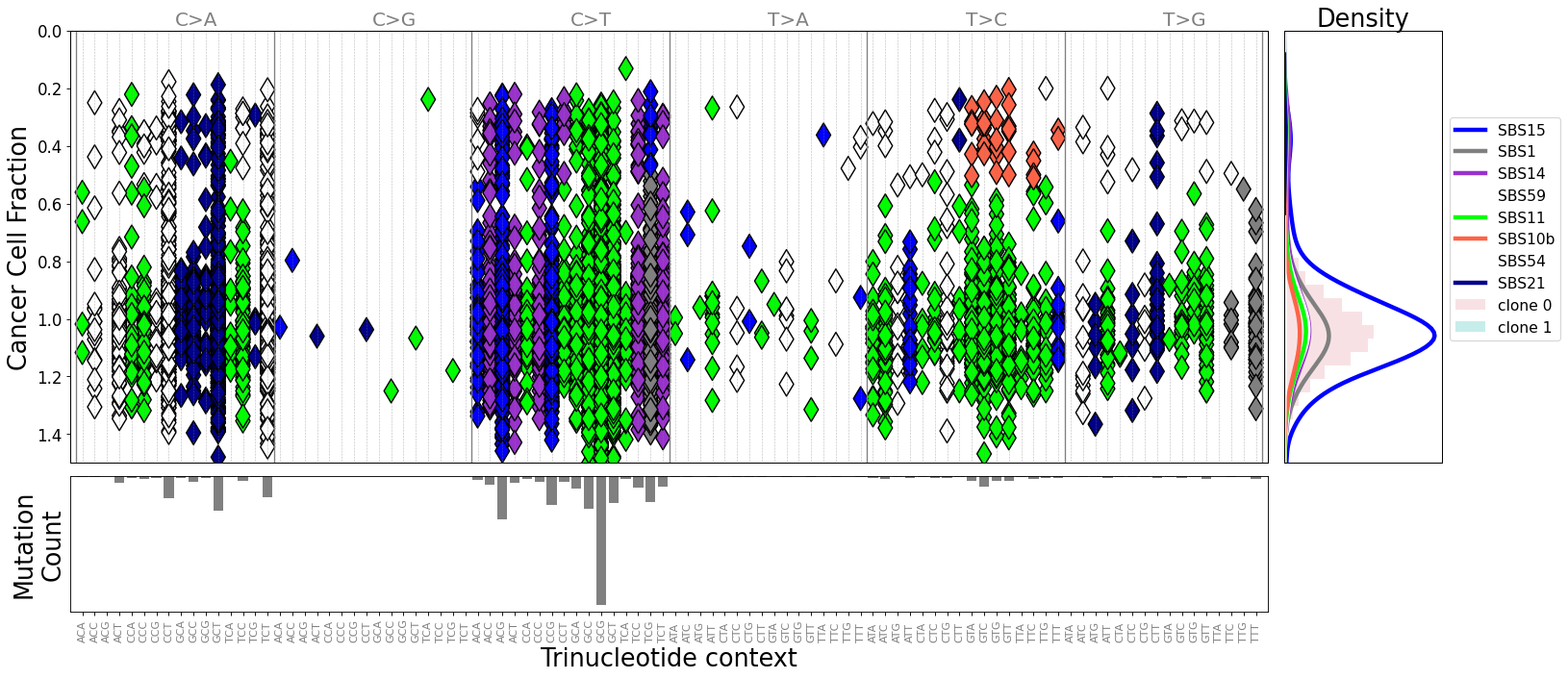


Pt24


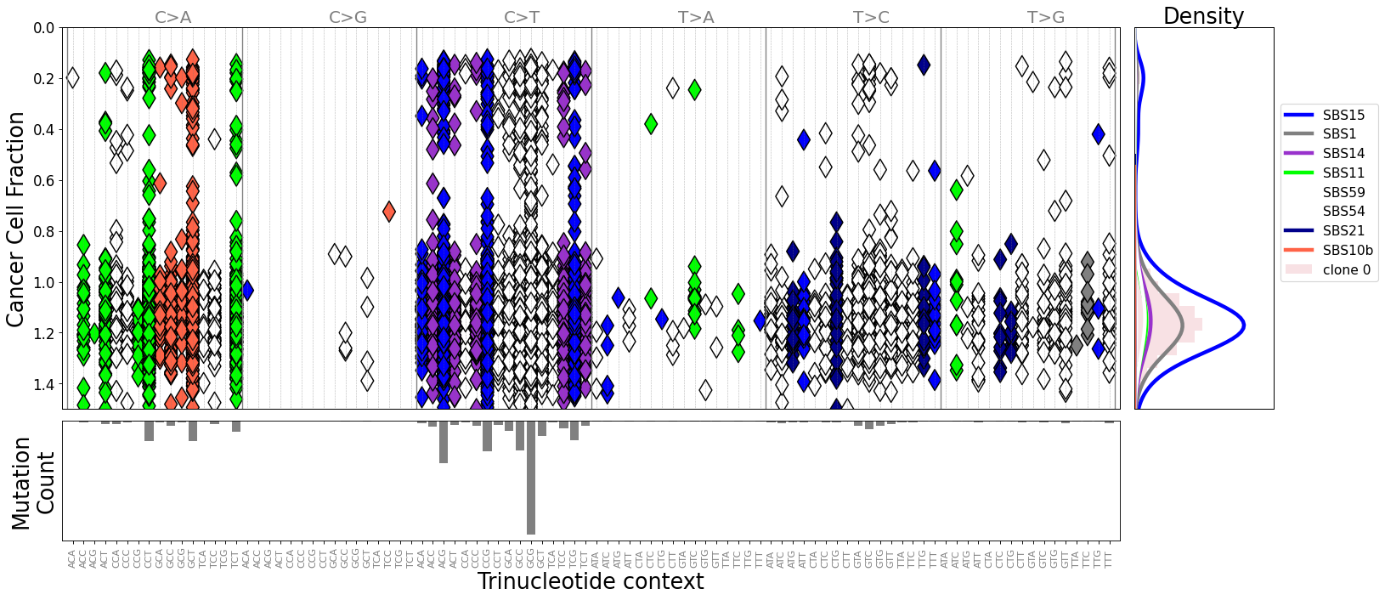


Pt30


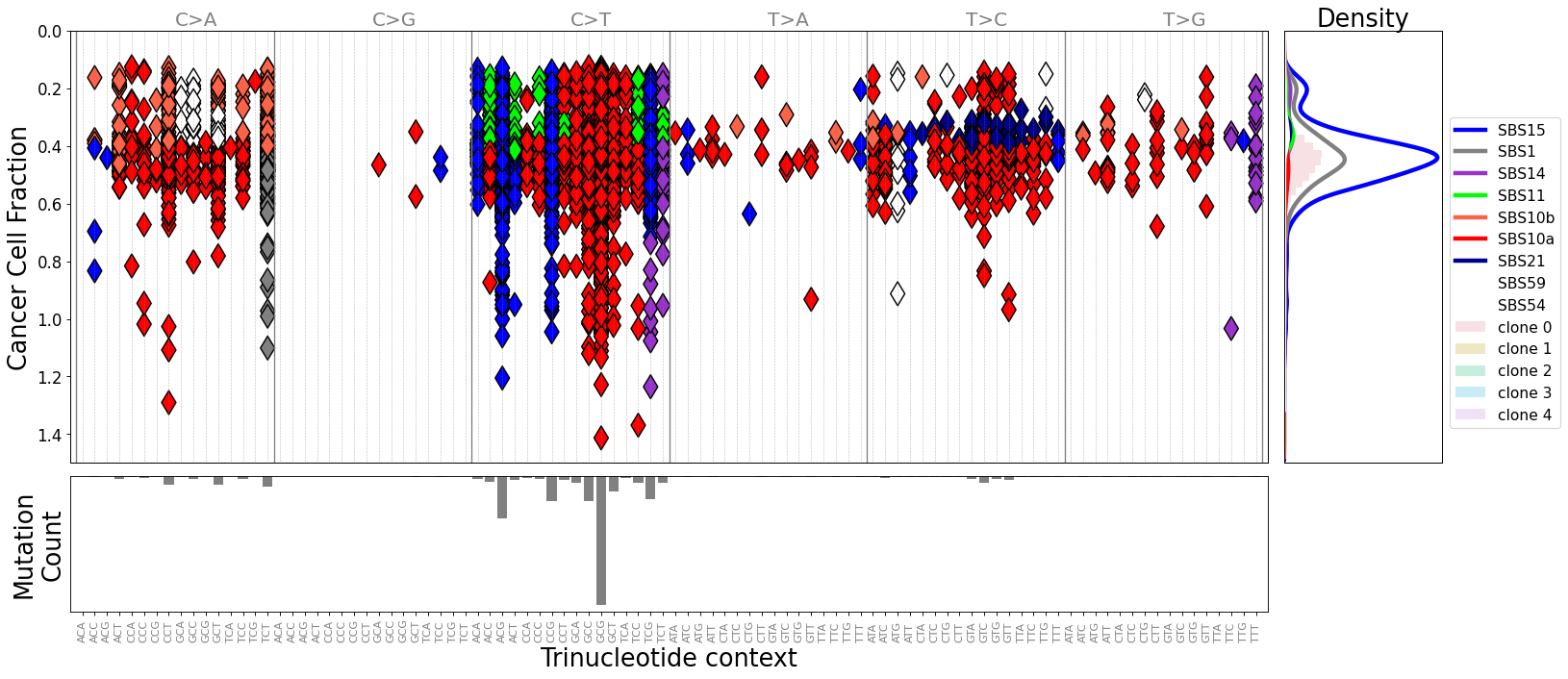

Pt19


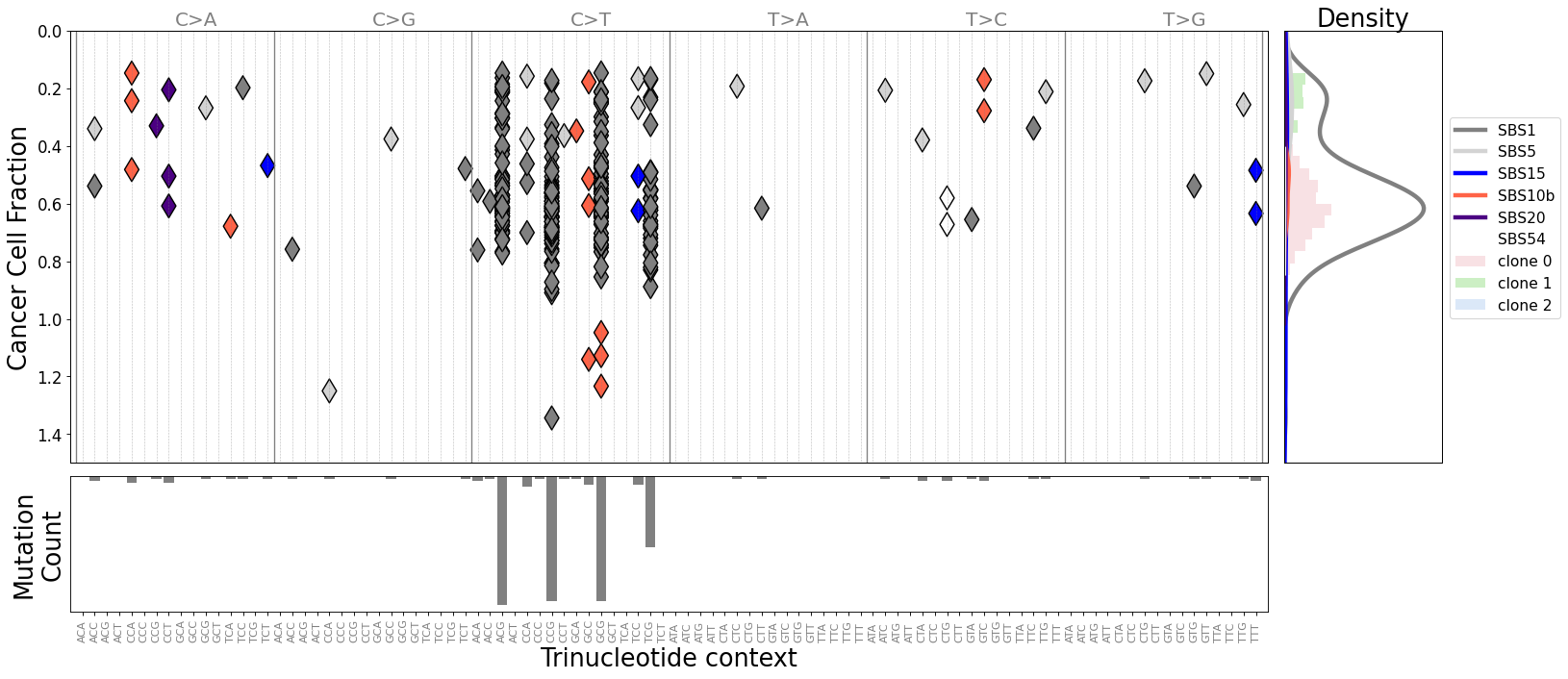

Pt4B

**Supplementary Figure S4: Mutational signature activity across the clones of each tumor**


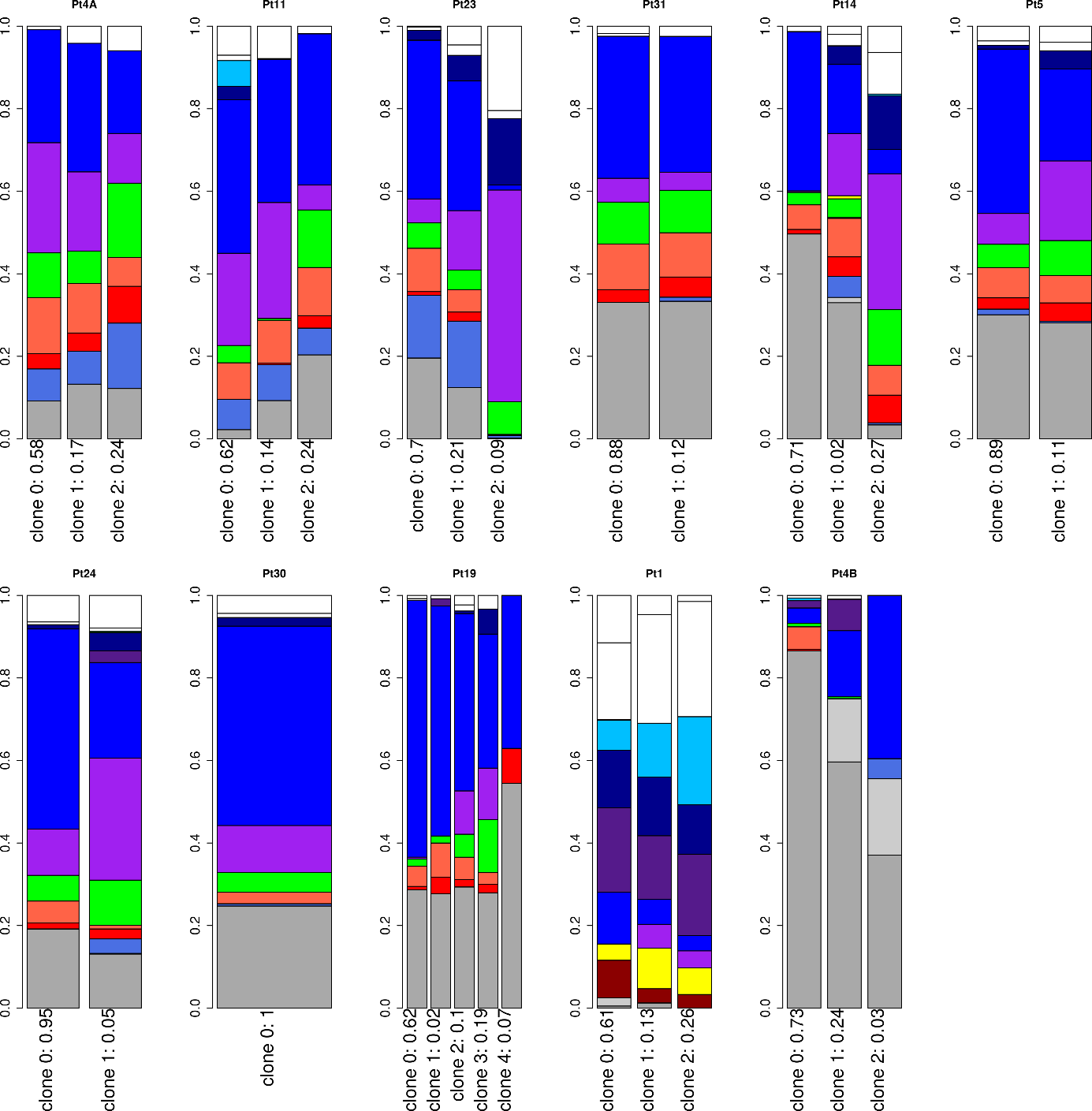
CloneSig provides several outputs, including the clone proportions and the signature activity of each clone. For each tumor, the signature activity of each mutational signature is shown in every clone. Clone proportions are indicated for each clone for all patients.
