# Supplementary material for: Glioma oncogenesis in the Constitutional mismatch repair deficiency (CMMRD) syndrome
Source: Neurooncol Adv. 2024 Jul 11;6(1):vdae120. doi: 10.1093/noajnl/vdae120 (PMC11372297; doi:10.1093/noajnl/vdae120)
Supplement: vdae120_suppl_Supplementary_Data [file vdae120_suppl_Supplementary_Data.zip › suppl/SupplementaryMethods_CMMRD_V11.docx]

**Supplementary Methods**

**Guerrini-Rousseau L & Merlevede J**

**I - Supplementary Materials and Methods**

**Patient samples.** We performed whole exome sequencing of paired normal tumor for 12 patients at diagnosis or relapse.

**Library preparation and whole exome sequencing.** Genomic DNA is captured using Agilent in-solution enrichment methodology (SureSelect XT Clinical Reasearch Exome, Agilent) with their biotinylated oligonucleotides probes library, followed by paired-end 75 bases massively parallel sequencing on Illumina HiSeq4000. For detailed explanations of the process, see Gnirke publication in Nature Methods [S1]. Sequence capture, enrichment and elution are performed according to manufacturer’s instruction and protocols (SureSelect, Agilent) without modification except for library preparation performed with NEBNext® Ultra kit (New England Biolabs®). For library preparation 600 ng of each genomic DNA are fragmented by sonication and purified to yield fragments of 150-200 bp. Paired-end adaptor oligonucleotides from the NEB kit are ligated on repaired, a tailed fragments then purified and enriched by 8 PCR cycles. 1200ng of these purified Libraries are then hybridized to the SureSelect oligo probe capture library for 72 hr. After hybridization, washing, and elution, the eluted fraction is PCR-amplified with 9 cycles, purified and quantified by QPCR to obtain sufficient DNA template for downstream applications. Each eluted-enriched DNA sample is then sequenced on an Illumina HiSeq4000 as paired-end 75b reads. Image analysis and base calling is performed using Illumina Real Time Analysis (2.7.3) with default parameters. Quality of reads was evaluated using FastQC (<http://www.bioinformatics.bbsrc.ac.uk/projects/fastqc/>).

**Whole exome sequencing analysis.** Raw reads were filtered with Trimommatic 0.33 to remove adaptors, truncate any read whose average quality on a sliding window (6 bases) was ≤25, remove the start and the end of a read if ≤25 and any read with an average quality ≤25 or a length<40. Reads were aligned to the reference human genome hg19 (Genome Reference Consortium GRCh37) using BWA 0.7.12 (Burrows-Wheeler Aligner) mem algorithm [S2]  with default parameters. Reads with mapping quality<20 were excluded using samtools view -Sq 20. Sam files were sorted and converted into bam files using Picard ([http://picard.sourceforge.net](http://picard.sourceforge.net/)) version 1.134 SortSam. Polymerase chain reaction duplicates were removed with Picard MarkDuplicates. Local realignment around indels was performed using GATK 3.4.0 (Genome Analysis ToolKit)  [S3] RealignerTargetCreator and IndelRealigner using known indels from 1000G [S6]. Base quality score recalibration was performed using GATK BaseRecalibrator using also known indels from 1000G and SNPs from dbSNP144 [S13]. The mean coverage in the targeted regions was 151x in tumor samples and 90x in control samples (**Supplementary** **Table S1**). When converting realigned recalibrated bam files into mpileup files using samtools 1.2, reads and bases with a Phred-based quality score ≤20 were ignored.

**Somatic variants calling.** Somatic single nucleotide variants (SNVs) and indels were called with VarScan2 somatic 2.3.9 [S4] using –min-coverage-normal 10 and --min-coverage-tumor 8. Only variants with somatic p-value below 10^-3^ were reported. In addition to the Fisher Exact test of VarScan2, we required (variant allele frequency in the tumor sample - variant allele frequency in the normal sample) ≥5% to distinguish somatic from germline variations. Variants with variant allele frequency in the normal sample>=5% were excluded. Numbers of somatic variants are given in **Supplementary Table 2.**

**Germline variant calling.** Germline single nucleotide variants (SNVs) and indels were also called with VarScan2 somatic using –min-coverage-normal 4 and --min-coverage-tumor 4. Only variants with p-value below 10^-2^ and “Germline” somatic_status were reported.

We extracted in particular germline variants in PMS2, MSH2, MSH6 and PMS1 (and EPCAM) to identify which altered gene in the complex was responsible for CMMRD in each patient (**Supplementary Table 5**).

**Annotation.** Remaining variants were annotated with Annovar [S5] . The mutations were searched in dbSNP [S13], and Exome Sequencing Project [S7] (ESP5400). Conservation of the position was predicted by PhyloP [S8] and the effect of the mutation was predicted by SIFT, Polyphen2 [S10], LRT [S11] and MutationTaster [S12]. Somatic variants were searched in COSMIC [S16] and ClinVar [S17] databases.

**Mutational Signatures.**

SigProfilerAssignment (PMID: 37502962) version 0.1.0 was used to extract the mutational signatures acting in the patients in a supervised way, namely using the most recent COSMIC mutational signatures (v3.4 - October 2023) composed of 67 SBS signatures. Somatic coding (including synonymous) SNV were examined. Starting from the matrix of counts for each trinucleotide context and for each patient, SigProfilerAssignment extracted the SBS related signatures and output the contribution of each signature in each patient. It also provided statistics to compare the original spectrum of mutations to the reconstructed one.

SigProfilerAssignment was applied with default parameters. Solely the behavior of ‘background signatures’ was modified in the code. SBS1 and SBS5 are defined as ‘background signatures’ by default, i.e. they are extracted more easily than the other signatures, on the grounds of being more prevalent than the other signatures in tumors in general. Given that the cohort is composed of pediatric samples, this preference for SBS1 and SBS5 (age-related) did not seem appropriate. ‘background signatures’ were removed, thus SBS1 and SBS5 were treated like the other mutational signatures.

To evaluate the extracted mutational signatures and their contribution, we looked at the statistics comparing the original spectrum to the reconstructed one. Cosine similarity was higher than 0.96 for ten patients and around 0.90 for Pt1 and Pt26 in the global analysis. Cosine similarity was higher than 0.95 except for Pt1 in the analysis by bursts. Note that Pt 26 has only 71 mutations, which is a very low number to extract mutational signatures.

**Copy number variations and loss of heterozygosity.** We used Sequenza R package version 2.1.1 with default parameters to look for copy number variations and loss of heterozygosity. Using an in-house method (EaCoN (https://github.com/gustaveroussy/EaCoN)), we confirmed the copy number segmentation obtained with Sequenza.

The list of selected polymerases is given in **Supplementary Table 4.**

**Recurrently mutated genes before the polymerase alteration.** Coding SNV showing a higher VAF than the VAF of the mutated polymerase were selected. All coding SNV were selected for the patients with no POLE or POLD1 mutation. All coding SNV were combined, synonymous SNV were removed to keep only the variants with a potential “damaging” effect, and genes, with at least one coding (synonymous excluded) somatic SNV, mutated in at least 8 out 12 patients were reported. After removing genes that are reported as artifacts in sequencing analysis [S21], there remained 30 such genes that are represented in **Figure 5A** .

**II - Supplementary References**

S1. Gnirke, A. et al. Solution hybrid selection with ultra-long oligonucleotides for massively parallel targeted sequencing. Nat Biotechnol 27, 182-189 (2009).

S2. Li H, Durbin R. Fast and accurate long-read alignment with Burrows-Wheeler transform. Bioinformatics 2010;26:589-95.

S3. McKenna A, Hanna M, Banks E, et al. The Genome Analysis Toolkit: a MapReduce framework for analyzing next-generation DNA sequencing data. Genome Res 2010; 20:1297-303.

S4. Koboldt DC, Zhang Q, Larson DE, et al. VarScan 2: somatic mutation and copy number alteration discovery in cancer by exome sequencing. Genome Res 2012;22:568-76.

S5. Wang K, Li M, Hakonarson H. ANNOVAR: functional annotation of genetic variants from high-throughput sequencing data. Nucleic Acids Res 2010;38:e164.

S6. Kuehn, BM. 1000 Genomes Project promises closer look at variation in human genome. JAMA 2008;300:2715.

S7. Fu W, O'Connor TD, Jun G, et al. Analysis of 6,515 exomes reveals the recent origin of most human protein-coding variants. Nature 2013;493:216-20.

S8. Pollard KS, Hubisz MJ, Rosenbloom KR, Siepel A. Detection of nonneutral substitution rates on mammalian phylogenies. Genome Res 2010;20:110-21.

S9. Kumar P, Henikoff S, Ng PC. Predicting the effects of coding non-synonymous variants on protein function using the SIFT algorithm. Nat Protocols 2009;4:1073-81.

S10. Adzhubei I, Jordan DM, Sunyaev SR. Predicting functional effect of human missense mutations using PolyPhen-2. Curr Protoc Hum Genet 2013; 7: 7-20.

S11. Chun S, Fay JC, Identification of deleterious mutations within three human genomes. Genome Res 2009;19:1553-61.

S12. Schwarz JM, Rodelsperger C, Schuelke M, Seelow D, MutationTaster evaluates disease-causing potential of sequence alterations. Nature Methods 2010;7:575-6.

S13. Sherry ST, Ward MH, Kholodov M, et al. dbSNP: the NCBI database of genetic variation. Nucleic Acids Res 2001;29:308-11.

S16. Forbes SA, Bindal N, Bamford S, et al. COSMIC: mining complete cancer genomes in the Catalogue of Somatic Mutations in Cancer. Nucleic Acids Res 2011;39:945-50.

S17. Landrum MJ, Lee JM, Riley GR, et al. ClinVar: public archive of relationships among sequence variation and human phenotype. Nucleic Acids Res 2014;42: 980-5.

S21. Fuentes Fajardo KV, Adams D, NISC Comparative Sequencing Program, et al. Detecting false-positive signals in exome sequencing. Hum Mutat 2012;33:609-13.

S22. Job B, Dayris T. Segment’it: <https://github.com/gustaveroussy/EaCoN>

S23. Ingham D, Diggle CP, Berry I, et . Simple detection of germline microsatellite instability for diagnosis of constitutional mismatch repair cancer syndrome. Hum Mutat 2013:34:847-52.

S24. Bodo S, Colas C, Buhard O, et al. Diagnnosis of constitutional mismatch repair deficiency syndrome based on microsatellite instability and lymphocyte tolerance to methylating agents. Gastroenterology 2015;149:1017-29.

S25. Bouvet D, odo S, Munier A, et al. Methylation tolerance-based functional assay to assay variants of unknown significance in the MLH1 and MSH2 genes and identify patients with Lynnch syndrome. Gastroenterology 2019;157:421-31.

S26. Abecassis J, Reyal F, Vert JP. Clonesig can jointly infer intra-tumor heterogeneity and mutational signature activity in bulk tumor sequencing data. Nat Commun 2021;12:5352.
